# Synthesis and bioactivities evaluation of quinazolin-4(3*H*)-one derivatives as α-glucosidase inhibitors

**DOI:** 10.1186/s13065-022-00885-z

**Published:** 2022-11-15

**Authors:** Mahshid Moheb, Aida Iraji, Navid Dastyafteh, Minoo Khalili Ghomi, Milad Noori, Somayeh Mojtabavi, Mohammad Ali Faramarzi, Fatemeh Rasekh, Bagher Larijani, Kamiar Zomorodian, Seyed Esmaeil Sadat-Ebrahimi, Mohammad Mahdavi

**Affiliations:** 1 Department of Medicinal Chemistry, Faculty of Pharmacy and Pharmaceutical Sciences Research Center, Tehran University of Medical Sciences, Tehran, Iran; 2 Stem Cells Technology Research Center, Shiraz University of Medical Sciences, Shiraz, Iran; 3 Central Research Laboratory, Shiraz University of Medical Sciences, Shiraz, Iran; 4 Endocrinology and Metabolism Research Center, Endocrinology and Metabolism Clinical Sciences Institute, Tehran University of Medical Sciences, Tehran, Iran; 5 Department of Pharmaceutical Biotechnology, Faculty of Pharmacy, Tehran University of Medical Sciences, Tehran, Iran; 6 Department of Biology, Payame Noor University (PNU), Tehran, Iran; 7 Department of Medical Mycology and Parasitology, School of Medicine, Shiraz University of Medical Sciences, Shiraz, Iran

**Keywords:** Acetamide, α-Glucosidase inhibition, Molecular docking, Quinazolin-4(3*H*)-one

## Abstract

The development of new antidiabetes agents is necessary to obtain optimal glycemic control and overcome its complications. Different quinazolin-4(3*H*)-one bearing phenoxy-acetamide derivatives (**7a–r**) were designed and synthesized to develop α-glucosidase inhibitors. All the synthesized derivatives were evaluated against α-glucosidase in vitro and among them, compound **7b** showed the highest α-glucosidase inhibition with an IC_50_ of 14.4 µM, which was ∼53 times stronger than that of acarbose. The inhibition kinetic studies showed that the inhibitory mechanism of compound **7b** was a competitive type towards α-glucosidase. Also, molecular docking studies analyzed the interaction between the most potent derivative and α-glucosidase. Current findings indicate the new potential of quinazolin-4(3*H*)-ones that could be used for the development of novel agents against diabetes mellitus.

**Supplementary Information:**

The online version contains supplementary material available at 10.1186/s13065-022-00885-z.

## Introduction

Diabetes mellitus (DM) has become one of the important issues in recent years with the rising obesity crisis categorized as 6th most frequent cause of global mortality [1]. DM can be classified into three major types: type I DM (insulin-dependent) due to immune-mediated β cells destructions; type II DM (non-insulin-dependent) due to an insulin secretory defect and insulin resistance as well as gestational diabetes that develops during pregnancy [2]. T2DM accounts for around 90–95% of the diabetic population and has become a major health problem worldwide [3]. All three types of DM are characterized by chronic high glucose levels which stimulate the generation of ROS that has a pivotal role in diabetic complications [4] Reducing postprandial hyperglycemia could prevent and minimize the risk of micro-and macro-vascular complications [5, 6].

In this context, inhibiting the activities of carbohydrate digestive enzymes is considered effective management of T2DM to retard glucose absorption [7]. α-glucosidase is known as a key enzyme responsible for the digestion of carbohydrates situated in the brush border of the small intestine of humans so that the di- and oligosaccharides undergo hydrolysis to glucose for intestinal absorption [8, 9].

Acarbose, miglitol, and voglibose are important anti-diabetic drugs used in clinical treatment as hypoglycemic compounds [5], but the efficacy of these is drugs a matter of debate due to unwanted side effects including flatulence, meteorism, abdominal distention, and the possibility of diarrhea [10, 11]. Also, it was reported that diabetic patients can develop resistance to current regimens. As a consequence, novel inhibitors are required to improve DM treatment. Numerous studies have described the inhibition of α-glucosidase induced by triazole [12], chromene [12], pyridine [13], quinolone [3], cyanoacetohydrazide [7], and benzimidazole [14] based compounds.

Quinazoline is a fused heterocyclic system and a core structure in a large variety of compounds that exhibited diverse biological activities, including anti-cancer, anti-microbial, anti-tubercular, anti-melanogenesis and anti-convulsant activities [15, 16]. Furthermore, recent studies demonstrated the α-glucosidase inhibitory activity of quinazoline-based derivatives [17, 18]. In this regard, substituted quinazolin-4(3*H*)-one derivative linked to coumarin (**A**) nucleus exhibited promising inhibition. SARs showed that quinazolinone derivatives lacking a coumarin ring reduce inhibitory activity; however, quinazolinone-coumarin hybrids significantly increase the inhibitory activities confirming the substitution on the quinazoline ring improved the potencies [19]. Quinazolinone-1,2,3-triazole derivatives (compound **B**) were exhibited as potent α-glucosidase inhibitors with no toxicity against breast cancer cell line MCF-7 [20]. Analog **C** with phenoxy-quinazolinone pharmacophore is another potent competitive α-glucosidase inhibitor with *K*_*i*_ value of 44 µM [21].

**Fig. 1:**
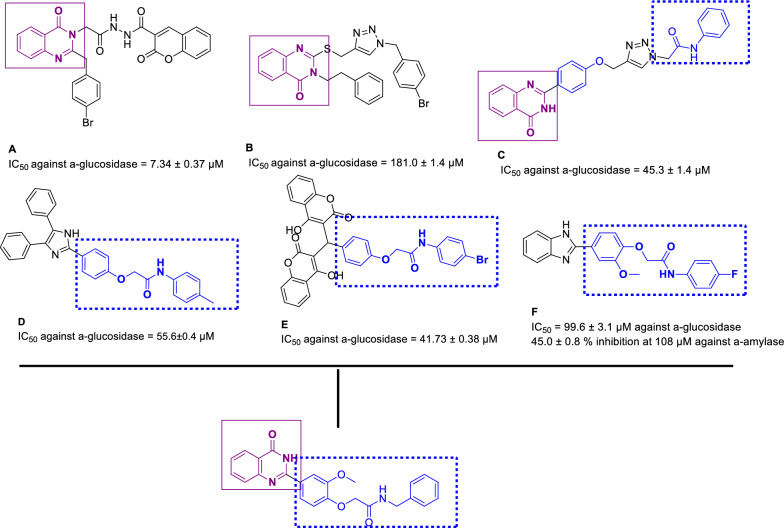
Rational design of new quinazolin-4(3*H*)-one bearing aryl substituted derivatives as potent α-glucosidase inhibitors based on previously reported pharmacophore active units

Recently, it was shown that aryl-substituted phenoxy-acetamide scaffolds were reported as potent α-glucosidase inhibitors. C=O moiety of acetamide as the key skeletons anchoring can stabilize α-helices, β-sheets, and other secondary structures of biological macromolecules through participation in various forms of interaction including hydrogen bonding, nucleophile–carbonyl, carbonyl–carbonyl (CO/CO) interaction [22]. Assessments on diphenylimidazole core attached to the various N-aryl acetamides (Fig. 1, Compound **D**) showed higher inhibitory activities with IC_50_ values of 55.6–149.2 µM than the activity of acarbose. In silico study exhibited several hydrophobic and H-bound interactions interaction with the binding site [23]. Also, phenoxybiscoumarin linked to different phenyl acetamides (Fig. 1, Compound **E**) was reported as an α-glucosidase inhibitor with an IC_50_ value of 41.73 to > 750 μM compared with acarbose (IC_50_ = 750.0 μM). Interestingly, the critical role of acetamide moiety was confirmed via H-bound interaction with the active site of the enzyme [24]. In our previous study novel series of benzimidazole-bearing phenoxy acetamide derivatives were developed as α-glucosidase inhibitors with IC_50_ values between 99.6 ± 3.1 to > 750 μM. In silico studies showed that the phenoxy linker of potent inhibitor exhibited H-bound interaction with Asp616 and/or Asp282 [14].

Keeping in view of the importance of quinazolin-4(3*H*)-one ring and the phenoxy-acetamide moiety to establish different forms of interactions with the α-glucosidase binding site as well as provide a suitable site for derivitization to evaluate the SARs, this work focused on the design and synthesis of quinazolin-4(3*H*)-one bearing aryl substituted phenoxyacetamide derivatives. The inhibition of all derivatives against α-glucosidase as well as kinetic studies was performed to describe the inhibition pattern. In silico studies were also considered to gain a better understanding of the interactions of the most potent derivative with the α-glucosidase binding site.

## Results and discussions

### Chemistry

**Scheme 1.**
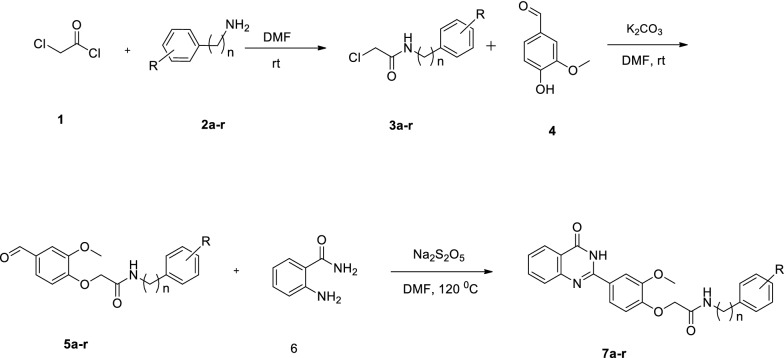
Synthesis of compounds **7a–r**

The synthetic route was described in Scheme 1.

To a solution of aniline derivatives in DMF, chloroacetylchloride was added and the mixture was stirred at room temperature for 5 h to get the desired product, **3a–r**. The reaction of 4-hydro-3-methoxy benzaldehyde (**4**) compound **3a–r** in DMF in the presence of potassium carbonate gave compound **5a–r**. Finally, 2-aminobenzamide **6** reacted with different aldehydes, **5a–r** in DMF in the presence of Na_2_S_2_O_5_ at room temperature to prepare desired derivative **7a–r**. The molecular structure of all compounds was deduced by spectroscopic techniques including EI-MS, CNHOS, ^1^H, and ^13^C-NMR.

### α-glucosidase inhibitory activity

Derivatives **7a–r** were synthesized to evaluate their potency as α-glucosidase inhibitors. Results are summarized in Table 1 in terms of IC_50_s.

**Table 1:** α-Glucosidase inhibitory activity of compounds **7a–r**


Compounds	R	IC_50_ (µM)^a^	Concentrations of precipitation (µM)
**7a**		363.4 ± 1.1	> 150
**7b**		14.4 ± 0.2	> 150
**7c**		255.5 ± 1.7	> 150
**7d**		692.5 ± 2.4	> 150
**7e**		114.3 ± 1.8	> 150
**7f**		25.6 ± 1.0	> 150
**7g**		332.9 ± 3.1	> 150
**7h**		454.83 ± 1.8	> 150
**7i**		326.2 ± 2.3	> 150
**7j**		715.4 ± 1.8	> 150
**7k**		529.6 ± 0.6	> 150
**7l**		> 750	> 150
**7m**		> 750	> 150
**7n**		> 750	> 150
**7o**		> 750	> 150
**7p**		> 750	> 150
**7q**		> 750	> 150
**7r**		595.0 ± 3.1	> 150
**Acarbose**^**b**^		750.0 ± 1.6	

^a^Data presented here are the mean ± S.E of three independent experiments

^b^Positive control

**7a** as unsubstituted derivative exhibited an IC_50_ value of 363.4 µM. Compounds **7b–g** were solely substituted with halogen groups including F, Cl, and Br, at a different position of the phenyl ring. Mostly, these analogs displayed improved inhibition potential against the α-glucosidase enzyme. Amongst, compound **7b** with *ortho* fluorine substitution was found to be the most potent α-glucosidase inhibitor with IC_50_ values of 14.4 ± 0.2 µM. A comparison of compound **7b** with compound **7c** showed the positive effect of *ortho*-fluorine on inhibitory potential *vs para*-fluorine counterparts. Vice versa trends were seen in chlorine substitutions so that potencies changed in the following order: *para*-chlorine (**7f**, IC_50_ = 25.6 µM) > *meta*-chlorine (**7e**, IC_50_ = 114.3 µM) > *ortho*-chlorine (**7d** IC_50_ = 692.5 µM).

In the case of halogen substitutions, it can be understood that the position of the halogen played the most dominant role in the potency so that *ortho*-fluorine followed by *para*-chlorine group demonstrated high inhibitory potential.

Compounds **7h–j** bearing methyl substitutions at different positions of phenyl part were found to have fewer inhibitory potential compared to **7a** as unsubstituted counterpart. The exception in this trend came back to **7i** with almost similar potency as **7a**. Less inhibitory potential of compounds **7j** than compounds **7h** and **7i** might be due to the steric effect by the second methyl-substituted position. Again as can be seen in **7k** bearing ethyl substitution, the increased bulkiness at the *para* position and inferior to the potency.

Compounds **7l**, **7m,** and **7n** containing methoxy, hydroxy, and nitro phenyl substitutions did not show any activity against α-glucosidase under the tested concentration. In these cases, it seems that even the presence of heteroatom with potency to participate in various form of interactions were unable to improve the inhibition.

Ring replacement of phenyl with naphthyl (**7o**) due to bigger size was found to completely inferior the inhibition. As can be seen in **7p**, **7q,** and **7r** the elongation of the linker (= 1) showed a deteriorated involvement of length in the inhibitory potential compared to their computer parts, **7a**, **7b,** and **7i**. This comparison showed that the shorter chain is playing an imperative character in the inhibitory potential. Also mostly *para*-substitutions reduced the potency, the only exception in this trend came back to **7f**.

### Enzyme kinetic studies

**Fig. 2:**
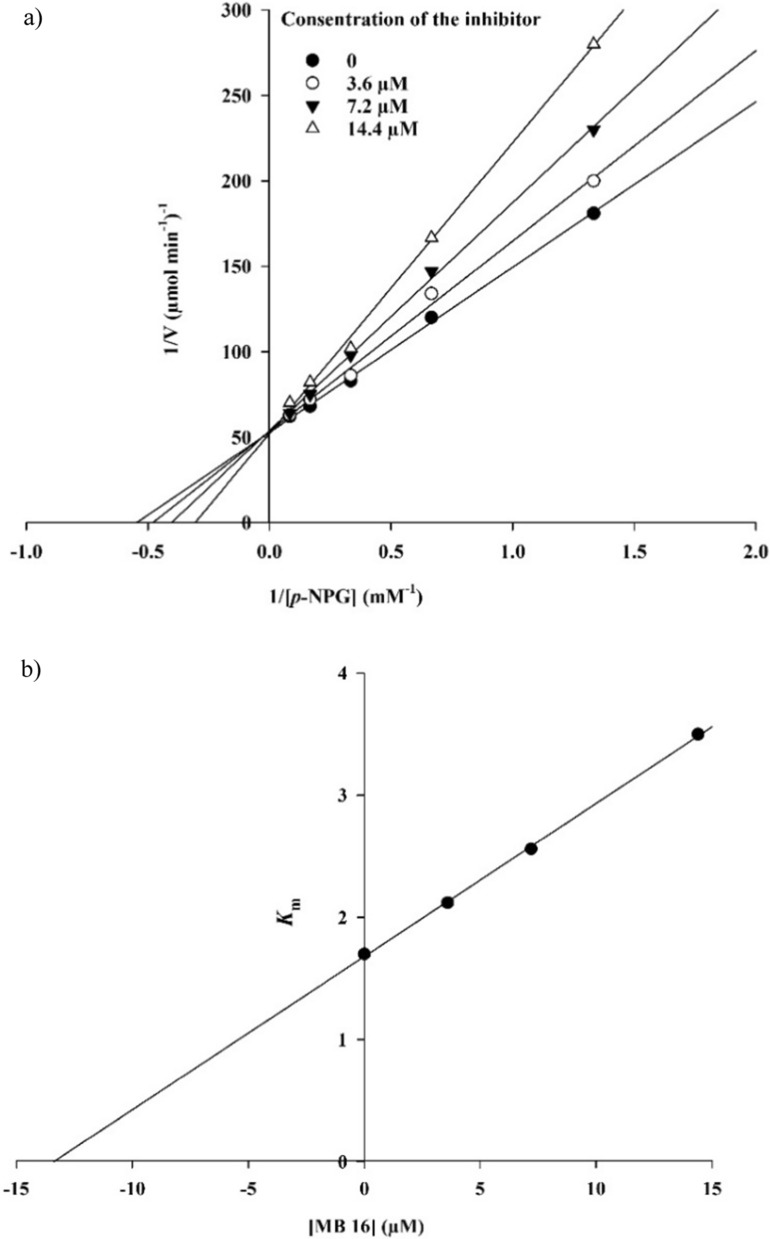
Kinetics of α-glucosidase inhibition by **7b**. **a** The Lineweaver–Burk plot in the absence and presence of different concentrations of the **7b**; **b** The secondary plot between *K*_m_ and various concentrations of the **7b**

According to Fig. 2a, the Lineweaver–Burk plot showed that the *K*_m_ gradually increased and *V*_*max*_ remained unchanged with increasing inhibitor concentration indicating a competitive inhibition. The results show sample **7b** binds to the active site on the enzyme and competes with the substrate for binding to the active site. Furthermore, the plot of the *K*_m_ versus different concentrations of inhibitor gave an estimate of the inhibition constant, *K*_i_ of 14.0 µM (Fig. 2b).

### Molecular docking

**Fig. 3.**
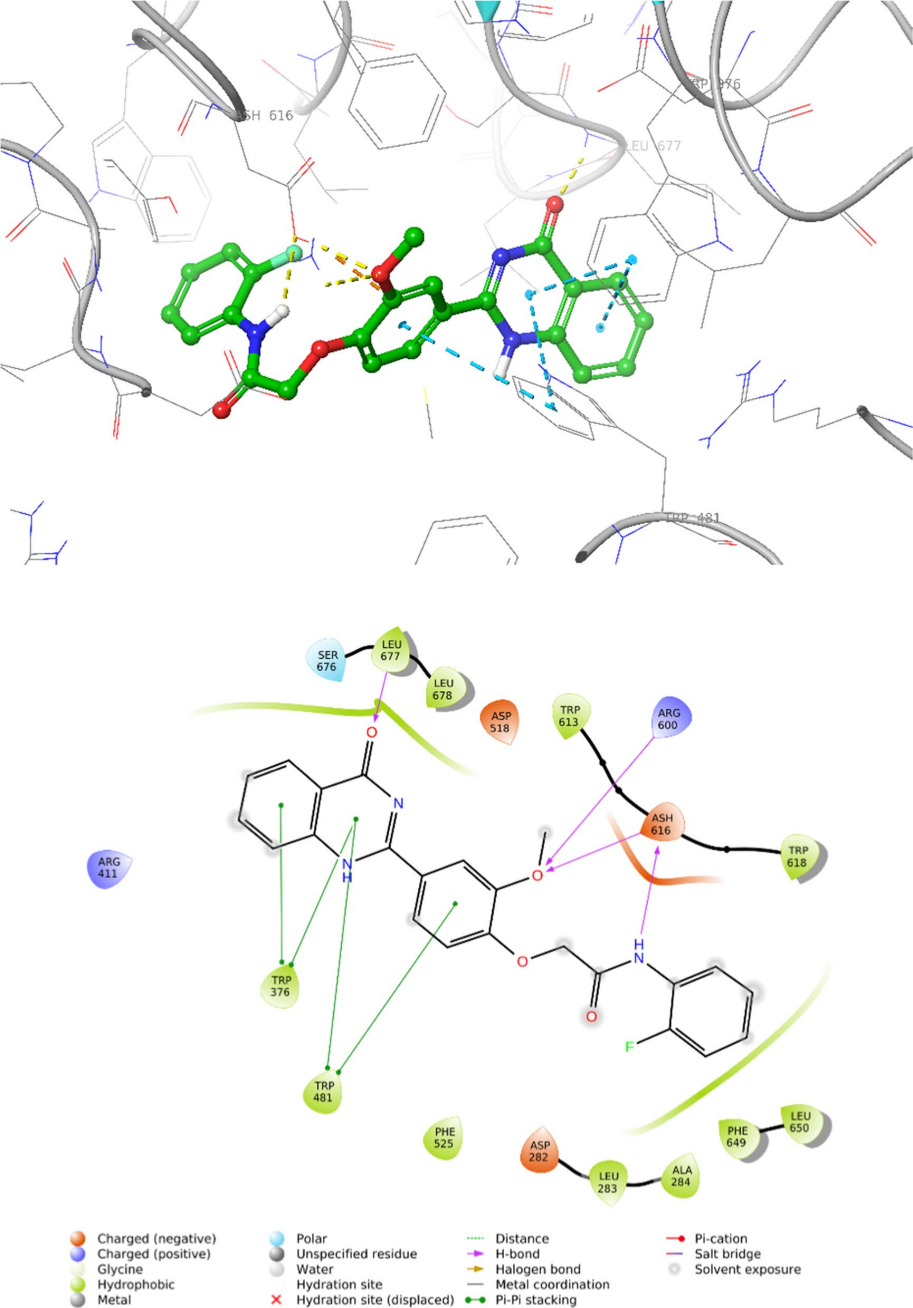
3D and 2D diagram of compound **7b** within the binding pocket of α-glucosidase

In order to clarify the interactions of the **7b** in the active site of α-glucosidase and explain the related inhibitory activities, molecular docking studies were performed. First, the molecular docking validation was executed on acarbose as a native ligand against the α-glucosidase and the alignment of the best pose of acarbose in the active site of the enzyme and crystallographic ligand recorded an RMSD value less than 2 Å which confirms the accuracy of docking. As observed in Fig. 3, quinazolin-4(3*H*)-one ring stabilized through participation in one H-bound with Leu677 plus three pi-pi stacking interactions with Trp346 and Trp481. On the other side of the molecule, another H-bound interaction was seen between acetamide moiety and Ash616. 2-methoxyphenoxy made two H-bound interactions with Arg600 and Ash606 as well as one hydrophobic interaction with Trp481.

## Conclusion

In summary, quinazolin-4(3*H*)-one derivatives (**7a–r**) were synthesized and evaluated for inhibitory activities against α-glucosidase. The synthesized derivatives showed a diverse range of inhibitory activities against α-glucosidase with IC_50_ values in the range of 14.4 ± 0.2 to > 750 µM. Compound **7b** recorded the highest inhibitory activity against α-glucosidase with around ~ 53 times better potencies than acarbose. The enzyme inhibitory kinetics and mode of binding for the most active inhibitor **7b** was performed which showed that the compound is a competitive inhibitor and effectively inhibits the target enzyme by binding to its active site. Docking results of the most active compound **7b** showed a good protein−ligand interaction profile against the corresponding target.

In summary, we believe that the search for new modifications of quinazolin-4(3*H*)-one will contribute to the development of potent anti-DM agents.

## Experimental section

### General

All chemicals and reagents were purchased from Merck and Aldrich. The IR spectra were obtained on a Nicolet Magna FTIR 550 spectrometer (potassium bromide disks). Melting points were determined using Kofler hot stage apparatus and are uncorrected. NMR spectra were recorded on a Bruker 300 MHz.

### Synthesis of compound 7a–r

To a solution of aniline derivatives (1 mmol) in DMF (4 mL), chloroacetylchloride (1.2 mmol) was added at 0 °C. The mixture was stirred at room temperature for 5 h and poured into water and then filtered to get the desired products (**3a–r**). The obtained solids were then filtered, dried, and recrystallized from ethanol. A 25 mL round bottom flask was charged with 4-hydro-3-methoxy benzaldehyde (1 mmol) and DMF (5 mL). 2-chloro-N-phenyl acetamide derivatives (1.1 mmol) were added, followed by potassium carbonate (1.2 mmol). The reaction mixture was stirred at room temperature for 5 h and then poured into ice water (25 ml). The products, **5a–r**, was collected by filtration and rinsed with water. **5a–r** derivatives (1 mmol) and 2-aminobenzamide (**6**, 1.2 mmol) were dissolved in 2 mL DMF and under stirring at room temperature, 1 mmol of sodium metabisulfite was added and allowed to react at 120 °C for about 4 h. After completion of the reaction, the mixture was precipitated in ice water, filtered, and dried at room temperature (Additional file 1).

#### 2-(2-methoxy-4-(4-oxo-3,4-dihydroquinazolin-2-yl)phenoxy)-N-phenylacetamide (7a)

Cream solid; Yield: 77%; MP = 192–194 °C; IR (KBr, v_max_) 3252 (NH), 3025 (CH Aromatic), 2970(CH Aliphatic), 1660 (C=O) Cm^−1^; ^1^H NMR (300 MHz, DMSO-*d*_*6*_) *δ* 12.44 (s, 1H, NH Quinazolinone), 10.14 (s, 1H, NH_Amide_), 8.14 (d, *J* = *8.20 Hz*, 1H, H_5_), 7.62 (s, 1H, H_2′_) 7.84–7.73 (m, 2H, H_6″_ H_7_), 7.70 (d, *J* = *8.00 Hz*, 1H, H_8_), 7.65 (d, *J* = 8.00 Hz, 2H, H_2″_, H_6″_), 7.45 (t, *J* = 7.60 Hz, 1H, H_6_), 7.32 (t, *J* = 7.80 Hz, 2H, H_3″_, H_5″_), 7.13–7.06 (m, 2H, H_5′_, H_4″_), 4.82 (s, 2H, CH_2_), 3.92 (s, 3H, OCH_3_) ppm. ^13^C NMR (75 MHz, DMSO-*d*_*6*_): *δ* 166.2, 162.3, 151.7, 150.1, 148.8, 148.7, 138.3, 134.3, 128.8, 127.4, 126.1, 125.8, 123.6, 121.0, 120.7, 119.5, 113.1, 111.0, 67.9, 55.7; ESI–MS (C_23_H_19_N_3_O_4_): calculated m/z 401.14 [M + H]^+^, observed m/z 401.21 [M + H]^+^; Anal. Calcd:C_23_H_19_N_3_O_4_; C, 68.82; H, 4.77; N, 10.47; Found; C, 68.98; H, 4.92; N, 10.65.

#### N-(2-fluorophenyl)-2-(2-methoxy-4-(4-oxo-3,4-dihydroquinazolin-2-yl)phenoxy)acetamide (7b)

Brown solid; Yield: 69%; MP = 222–225 °C; IR (KBr, v_max_) 3376 (NH), 3045 (CH Aromatic), 2995 (CH Aliphatic), 1674 (C=O) Cm^−1^; ^1^H NMR (300 MHz, DMSO-*d*_*6*_) *δ* 12.46 (s, 1H, NH Quinazolinone), 9.84 (s, 1H, NH_Amide_), 8.13 (d, *J* = *7.80 Hz*, 1H, H_5_), 7.98 (d, *J* = 6.60 Hz, 1H, H_6″_), 7.90–7.74 (m, 3H, H_7_, H_2′_, H_6′_), 7.70 (d, *J* = 8.0 1H, H_8_), 7.47 (t, *J* = *7.4 Hz*, 1H, H_6_), 7.33–7.24 (m, 1H, H_5′_), 7.22–7.04 (m, 3H, H_3″_, H_4″_, H_5″_), 4.89 (s, 2H, CH_2_), 3.93 (s, 3H, OCH_3_) ppm. ^13^C NMR (75 MHz, DMSO-*d*_*6*_): *δ* 166.6, 162.3, 153.4 (d, ^1^*J*_CF_ = 243.7 Hz), 151.6, 149.9, 148.8, 148.7, 134.3, 127.5, 125.8, 125.5, 125.3, 124.5, 123.4, 120.7, 115.6, 113.3, 111.2, 110.9, 67.5, 55.4; ESI–MS (C_23_H_18_FN_3_O_4_): calculated m/z 419.13 [M + H]^+^, observed m/z 419.18 [M + H]^+^; Anal. Calcd: C_23_H_18_FN_3_O_4_; C, 65.87; H, 4.33; N, 10.02; Found; C, 66.05; H, 4.58; N, 10.19.

#### N-(4-fluorophenyl)-2-(2-methoxy-4-(4-oxo-3,4-dihydroquinazolin-2-yl)phenoxy)acetamide (7c)

Brown solid; Yield: 67%; MP = 216–218 °C; IR (KBr, v_max_) 3369 (NH), 3030 (CH Aromatic), 2980 (CH Aliphatic), 1680 (C=O) Cm^−1^; ^1^H NMR (300 MHz, DMSO-*d*_*6*_) *δ* 12.45 (s, 1H, NH Quinazolinone), 10.21 (s, 1H, NH_Amide_), 8.13 (d, *J* = *7.70 Hz*, 1H, H_5_), 7.87–7.74 (m, 3H, H_7_, H_2′_, H_6′_), 7.73–7.60 (m, 3H, H_8_, H_2″_, H_6″_), 7.24 (t, *J* = *7.5 Hz*, 1H, H_6_), 7.20–7.11 (m, 2H, H_3″_, H_5″_), 7.10–7.05 (m, 1H, H_3″_, H_5″_), 4.87 (s, 2H, CH_2_), 3.93 (s, 3H, OCH_3_) ppm. ^13^C NMR (75 MHz, DMSO-*d*_*6*_): *δ* 166.1, 162.3, 158.2 (d, ^1^*J*_CF_ = 239.2 Hz), 151.7, 150.1, 148.8, 148.7, 134.7, 127.5, 125.8, 121.4, 121.0, 120.7, 115.5, 113.3, 111.3, 111.0, 67.8, 55.4; ESI–MS (C_23_H_18_FN_3_O_4_): calculated m/z 419.13 [M + H]^+^, observed m/z 419.24 [M + H]^+^; Anal. Calcd: C_23_H_18_FN_3_O_4_; C, 65.87; H, 4.33; N, 10.02; Found; C, 66.11; H, 4.54; N, 10.25.

#### N-(2-chlorophenyl)-2-(2-methoxy-4-(4-oxo-3,4-dihydroquinazolin-2-yl)phenoxy)acetamide (7d)

Brown solid; Yield: 78%; MP = 207–209 °C; IR (KBr, v_max_) 3357 (NH), 3070 (CH Aromatic), 2980 (CH Aliphatic), 1677 (C=O) Cm^−1^; ^1^H NMR (300 MHz, DMSO-*d*_*6*_) *δ* 12.40 (s, 1H, NH Quinazolinone), 9.53 (s, 1H, NH_Amide_), 8.13 (d, *J* = *7.80 Hz*, 1H, H_5_), 8.06 (d, *J* = 8.60 Hz, 1H, H_6″_), 7.87 (s, 1H, H_2′_), 7.86–7.77 (m, 2H, H_6′_, H_7_), 7.71 (d, *J* = *8.10 Hz*, 1H, H_8_), 7.59–7.42 (m, 2H, H_3″_, H_5″_), 7.36 (t, *J* = 7.70 Hz, 1H, H_6_), 7.22–7.11 (m, 2H, H_5′_, H_4″_), 4.87 (s, 2H, CH_2_), 3.94 (s, 3H, OCH_3_) ppm. ^13^C NMR (75 MHz, DMSO-*d*_*6*_): *δ* 166.4, 162.3, 151.7, 151.6, 149.4, 149.2, 148.7, 134.7, 134.7, 134.0, 132.5, 129.5, 127.7, 126.1, 125.8, 124.6, 122.4, 121.0, 10.7, 67.6, 55.6; Anal. Calcd: C_23_H_18_ClN_3_O_4_; C, 63.38; H, 4.16; N, 9.64; Found; C, 63.59; H, 4.41; N, 9.87.

#### N-(3-chlorophenyl)-2-(2-methoxy-4-(4-oxo-3,4-dihydroquinazolin-2-yl)phenoxy)acetamide (7e)

Brown solid; Yield: 74%; MP = 203–205 °C; IR (KBr, v_max_) 3363 (NH), 3070 (CH Aromatic), 2980 (CH Aliphatic), 1681 (C=O) Cm^−1^; ^1^H NMR (300 MHz, DMSO-*d*_*6*_) *δ* 12.47 (s, 1H, NH Quinazolinone), 10.28 (s, 1H, NH_Amide_), 8.13 (d, *J* = *7.90 Hz*, 1H, H_5_), 7.89–7.79 (m, 4H, H_7_, H_2′_, H_6′_, H_2″_), 7.70 (d, *J* = 8.1* Hz*, 1H, H_8_), 7.75–7.41 (m, 2H, H_6″_, H_5″_), 7.35 (t, *J* = *8.10 Hz*, 1H, H_6_), 7.16–7.06 (m, 2H, H_4″_, H_5′_), 4.83 (s, 2H, CH_2_), 3.93 (s, 3H, OCH_3_) ppm. ^13^C NMR (75 MHz, DMSO-*d*_*6*_): *δ* 166.6, 162.3, 151.7, 150.0, 148.8, 148.7, 139.8, 133.1, 130.5, 125.7, 123.3, 120.7, 118.6, 113.2, 111.3, 110.9, 67.7, 55.4; ESI–MS (C_23_H_18_ClN_3_O_4_): calculated m/z 435.10 [M + H]^+^, observed m/z 435.19[M + H]^+^; Anal. Calcd: C_23_H_18_ClN_3_O_4_; C, 63.38; H, 4.16; N, 9.64; Found; C, 63.57; H, 4.32; N, 9.81.

#### N-(4-chlorophenyl)-2-(2-methoxy-4-(4-oxo-3,4-dihydroquinazolin-2-yl)phenoxy)acetamide (7f)

Brown solid; Yield: 79%; MP = 211–213 °C; IR (KBr, v_max_) 3378 (NH), 3065 (CH Aromatic), 2990 (CH Aliphatic), 1703 (C=O) Cm^−1^; ^1^H NMR (300 MHz, DMSO-*d*_*6*_) *δ* 12.42 (s, 1H, NH Quinazolinone), 10.29 (s, 1H, NH_Amide_), 8.13 (d, *J* = *7.90 Hz*, 1H, H_5_), 7.85 (s, 1H, H_2′_) 7.82–7.75 (m, 2H, H_6′_, H_7_), 7.74–7.62 (m, 3H, H_2″_, H_6″_, H_8_), 7.47 (t, *J* = *7.50 Hz*, 1H, H_6_), 7.37 (d, *J* = 8.30 Hz, 2H, H_3″_, H_5″_), 7.09 (d, *J* = 8.40 Hz, 1H, H_5′_), 4.81 (s, 2H, CH_2_), 3.92 (s, 3H, OCH_3_) ppm. ^13^C NMR (75 MHz, DMSO-*d*_*6*_): *δ* 166.3, 162.3, 151.7, 150.0, 148.8, 148.7, 137.3, 134.3, 128.7, 128.3, 127.5, 127.2, 125.8, 121.1, 120.7, 113.0, 111.0, 67.8, 55.4; ESI–MS (C_23_H_18_ClN_3_O_4_): calculated m/z 435.10 [M + H]^+^, observed m/z 435.23[M + H]^+^; Anal. Calcd: C_23_H_18_ClN_3_O_4_; C, 63.38; H, 4.16; N, 9.64; Found; C, 63.54; H, 4.31; N, 9.91.

#### N-(4-bromophenyl)-2-(2-methoxy-4-(4-oxo-3,4-dihydroquinazolin-2-yl)phenoxy)acetamide (7g)

Brown solid; Yield: 84%; MP = 203–205 °C; IR (KBr, v_max_) 3378 (NH), 3020 (CH Aromatic), 2885 (CH Aliphatic), 1661 (C=O) Cm^−1^; ^1^H NMR (300 MHz, DMSO-*d*_*6*_) *δ* 12.42 (s, 1H, NH Quinazolinone), 10.27 (s, 1H, NH_Amide_), 8.13 (d, *J* = *7.90 Hz*, 1H, H_5_), 7.85 (s, 1H, H_2′_) 7.83–7.73 (m, 2H, H_6′_, H_7_), 7.69 (d, *J* = *7.90 Hz*, 1H, H_8_), 7.61 (d, *J* = 8.50 Hz, 2H, H_2″_, H_6″_), 7.53–7.41 (m, 3H, H_3″_, H_5″_, H_6_), 7.09 (d, *J* = 8.40 Hz, 1H, H_5′_), 4.81 (s, 2H, CH_2_), 3.93 (s, 3H, OCH_3_) ppm. ^13^C NMR (75 MHz, DMSO-*d*_*6*_): *δ* 166.4, 162.3, 151.6, 150.0, 148.8, 148.7, 137.7, 134.3, 131.6, 127.2, 126.1, 125.8, 121.4, 120.7, 115.3, 113.2, 111.1, 67.9, 55.7; ESI–MS (C_23_H_18_BrN_3_O_4_): calculated m/z 479.05 [M + H]^+^, observed m/z 479.13 [M + H]^+^; Anal. Calcd: C_23_H_18_BrN_3_O_4_; C, 57.51; H, 3.78; N, 8.75; Found; C, 57.64; H, 3.96; N, 8.91.

#### 2-(2-methoxy-4-(4-oxo-3,4-dihydroquinazolin-2-yl)phenoxy)-N-(o-tolyl)acetamide (7h)

Cream solid; Yield: 76%; MP = 196–198 °C; IR (KBr, v_max_) 3238 (NH), 3035 (CH Aromatic), 2965 (CH Aliphatic), 1682 (C=O) Cm^−1^; ^1^H NMR (300 MHz, DMSO-*d*_*6*_) *δ* 12.44 (s, 1H, NH Quinazolinone), 9.37 (s, 1H, NH_Amide_), 8.14 (d, *J* = *7.70 Hz*, 1H, H_5_), 7.86 (s, 1H, H_2′_) 7.85–7.75 (m, 2H, H_6′_, H_7_), 7.71 (d, *J* = *8.00 Hz*, 1H, H_8_), 7.59 (d, *J* = 7.90 Hz, 1H, H_3″_), 7.48 (t, *J* = 7.50 Hz, 1H, H_6_), 7.27–7.05 (m, 4H, H_5′_, H_6″_, H_5″_, H_4″_), 4.83 (s, 2H, CH_2_), 3.93 (s, 3H, OCH_3_), 2.23 (s, 3H, CH_3_) ppm. ^13^C NMR (75 MHz, DMSO-*d*_*6*_): *δ* 166.1, 162.3, 151.6, 149.8, 148.8, 148.7, 135.5, 134.6, 130.7, 130.3, 127.5, 126.1, 125.8, 125.1, 123.9, 121.0, 120.7, 113.1, 11.0, 67.7, 55.5, 17.4; Anal. Calcd: C_24_H_21_N_3_O_4_; C, 69.39; H, 5.10; N, 10.11; Found; C, 69.54; H, 5.28; N, 10.24.

#### 2-(2-methoxy-4-(4-oxo-3,4-dihydroquinazolin-2-yl)phenoxy)-N-(p-tolyl)acetamide (7i)

Cream solid; Yield: 76%; MP = 199–201 °C; IR (KBr, v_max_) 3400 (NH), 3020 (CH Aromatic), 2975 (CH Aliphatic) 1701 (C=O) Cm^−1^; ^1^H NMR (300 MHz, DMSO-*d*_*6*_) *δ* 12.47 (s, 1H, NH Quinazolinone), 10.07 (s, 1H, NH_Amide_), 8.13 (d, *J* = *7.70 Hz*, 1H, H_5_), 7.84 (s, 1H, H_2′_) 7.83–7.74 (m, 2H, H_6′_, H_7_), 7.70 (d, *J* = *8.00 Hz*, 1H, H_8_), 7.53–7.43 (m, 3H, H_2″_, H_6″_, H_6_), 7.16–7.03 (m, 3H, H_3″_, H_5″_, H_5′_), 4.78 (s, 2H, CH_2_), 3.92 (s, 3H, OCH_3_), 2.72 (s, 3H, CH_3_) ppm. ^13^C NMR (75 MHz, DMSO*-d*_*6*_): *δ* 165.9, 162.3, 151.7, 150.1, 148.8, 148.7, 135.8, 134.3, 132.6, 129.2, 129.1, 127.1, 125.9, 125.8, 121.0, 120.7, 119.4, 113.1, 11.2, 67.8, 55.4, 20.4; ESI–MS (C_24_H_21_N_3_O_4_): calculated m/z 415.15 [M + H]^+^, observed m/z 415.19 [M + H]^+^; Anal. Calcd: C_24_H_21_N_3_O_4_; C, 69.39; H, 5.10; N, 10.11; Found; C, 69.62; H, 5.27; N, 10.30.

#### N-(2,6-dimethylphenyl)-2-(2-methoxy-4-(4-oxo-3,4-dihydroquinazolin-2-yl)phenoxy)acetamide (7j)

Cream solid; Yield: 68%; MP = 215–217 °C; IR (KBr, v_max_) 3235 (NH), 3030 (CH Aromatic), 2970 (CH Aliphatic), 1684 (C=O) Cm^−1^; ^1^H NMR (300 MHz, DMSO-*d*_*6*_) *δ* 12.44 (s, 1H, NH Quinazolinone), 9.46 (s, 1H, NH_Amide_), 8.18–8.08 (m, 1H, H_5_), 7.86 (s, 1H, H_2′_) 7.85–7.66 (m, 2H, H_6′_, H_7_), 7.55–7.41 (m, 1H, H_8_), 7.29–6.98 (m, 5H, H_3″_, H_6_, H_5′_, H_6″_, H_5″_, H_4″_), 4.85 (s, 2H, CH_2_), 3.92 (s, 3H, OCH_3_), 2.14 (s, 6H, 2 × CH_3_) ppm. ^13^C NMR (75 MHz, DMSO-*d*_*6*_): *δ* 164.4, 162.0, 151.6, 150.3, 149.8, 148.8, 138.9, 135.5, 133.1, 130.6, 127.6, 126.0, 120.3, 118.9, 116.9, 113.1, 110.4, 67.2, 54.6, 17.4; Anal. Calcd: C_25_H_23_N_3_O_4_; C, 69.92; H, 5.40; N, 9.78; Found; C, 70.12; H, 5.67; N, 9.91.

#### N-(4-ethylphenyl)-2-(2-methoxy-4-(4-oxo-3,4-dihydroquinazolin-2-yl)phenoxy)acetamide (7k)

Cream solid; Yield: 81%;MP = 195–197 °C; IR (KBr, v_max_) 3401 (NH), 3065 (CH Aromatic), 2950(CH Aliphatic), 1670 (C=O) Cm^−1^; ^1^H NMR (300 MHz, DMSO-*d*_*6*_) *δ* 12.44 (s, 1H, NH Quinazolinone), 10.03 (s, 1H, NH_Amide_), 8.13 (d, *J* = *7.70 Hz*, 1H, H_5_), 7.85 (s, 1H, H_2′_) 7.83–7.75 (m, 2H, H_6′_, H_7_), 7.70 (d, *J* = *8.00 Hz*, 1H, H_8_), 7.53 (d, *J* = 8.10 Hz, 2H, H_2″_, H_6″_), 7.47 (t, *J* = 7.60 Hz, 1H, H_6_), 7.14 (d, *J* = 8.10 Hz, 2H, H_3″_, H_5″_), 7.09 (d, *J* = 8.40 Hz, 1H, H_5′_), 4.79 (s, 2H, CH_2_), 3.93 (s, 3H, OCH_3_), 2.53 (d, *J* = 7.50 Hz, 2H, CH_2 Ethyl_), 1.13 (t, *J* = 7.50 Hz, 3H, CH_3 Ethyl_) ppm. ^13^C NMR (75 MHz, DMSO-*d*_*6*_): *δ* 165.9, 162.3, 151.7, 150.1, 148.8, 148.7, 139.1, 136.0, 134.6, 127.9, 127.4, 126.1, 125.7, 120.7, 119.5, 119.4, 113.2, 113.1, 111.3, 111.0, 67.9, 55.8, 27.6, 15.7, 15.5; Anal. Calcd: C_25_H_23_N_3_O_4_; C, 69.92; H, 5.40; N, 9.78; Found; C, 70.11; H, 5.57; N, 9.94.

#### 2-(2-methoxy-4-(4-oxo-3,4-dihydroquinazolin-2-yl)phenoxy)-N-(4-methoxyphenyl)acetamide (7l)

Cream solid; Yield: 88%;MP = 205–207 °C; IR (KBr, v_max_) 3253 (NH), 3030 (CH Aromatic), 2975 (CH Aliphatic), 1661(C=O) Cm^−1^; ^1^H NMR (300 MHz, DMSO-*d*_*6*_) *δ* 12.44 (s, 1H, NH Quinazolinone), 9.93 (s, 1H, NH_Amide_), 8.13 (d, *J* = *7.80 Hz*, 1H, H_5_), 7.85 (s, 1H, H_2′_) 7.77–7.82 (m, 2H, H_6′_, H_7_), 7.75 (d, *J* = *8.10 Hz*, 1H, H_8_), 7.54 (d, *J* = 8.50 Hz, 2H, H_2″_, H_6″_), 7.47 (t, *J* = 7.50 Hz, 1H, H_6_), 7.09 (d, *J* = 8.10 Hz, 1H, H_5′_), 6.89 (d, *J* = 8.55 Hz, 2H, H_3″_, H_5″_), 4.77 (s, 2H, CH_2_), 3.93 (s, 3H, OCH_3_), 3.71 (s, 3H, OCH_3_) ppm. ^13^C NMR (75 MHz, DMSO-*d*_*6*_): *δ* 165.6, 162.3, 155.5, 151.7, 150.1, 148.8, 148.7, 134.3, 131.4, 127.5, 126.1, 125.7, 121.0, 120.9, 120.7, 114.0, 113.8, 113.1, 111.0, 67.9, 55.2, 55.0; Anal. Calcd: C_24_H_21_N_3_O_5_; C, 66.81; H, 4.91; N, 9.74; Found; C, 66.97; H, 5.03; N, 9.87.

#### N-(4-hydroxyphenyl)-2-(2-methoxy-4-(4-oxo-3,4-dihydroquinazolin-2-yl)phenoxy)acetamide (7m)

Cream solid; Yield: 67%; MP = 219–221 °C;; IR (KBr, v_max_) 3321(NH), 3035 (CH Aromatic), 2960 (CH Aliphatic), 1683 (C=O) Cm^−1^; ^1^H NMR (300 MHz, DMSO-*d*_*6*_) *δ* 12.46 (s, 1H, NH Quinazolinone), 9.13 (s, 1H, NH_Amide_), 8.13 (d, *J* = *7.3 Hz*, 1H, H_5_), 7.94–7.77 (m, 3H, H_7_, H_2′_, H_6’_) 7.70 (d, *J* = 7.7* Hz*, 1H, H_8_), 7.47 (t, *J* = *7.7 Hz*, 1H, H_6_), 7.40 (d, *J* = *8.1 Hz*, 2H, H_2″_, H_6″_), 7.07 (t, *J* = *8.1 Hz*, *1H*, H_5′_),6.71 (d, *J* = *8.3*, 2H, H_3″_, H_5″_), 4.75 (s, 2H, CH_2_), 3.92 (s, 3H, OCH_3_) ppm. ^13^C NMR (75 MHz, DMSO-*d*_*6*_): *δ* 165.4, 162.3, 153.6, 151.7, 150.1, 148.7, 139.1, 134.7, 129.9, 125.6, 120.6, 115.2, 114.9, 113.1, 110.8, 67.9, 55.4; Anal. Calcd: C_24_H_21_N_3_O_4_; C, 66.18; H, 4.59; N, 10.07; Found; C, 66.39; H, 4.76; N, 10.28.

#### 2-(2-methoxy-4-(4-oxo-3,4-dihydroquinazolin-2-yl)phenoxy)-N-(4-nitrophenyl)acetamide (7n)

Brown solid; Yield :81%; MP = 216–218 °C; IR (KBr, v_max_) 3343(NH), 3040(CH Aromatic), 2980(CH Aliphatic), 1674(C=O), 1560–1355(NO_2)_ Cm^−1^; ^1^H NMR (300 MHz, DMSO-*d*_*6*_) *δ* 12.49 (s, 1H, NH Quinazolinone), 9.57 (s, 1H, NH_Amide_), 8.26–8.19 (m, 2H, H_3″_, H_5″_), 8.12 (d, *J* = 7.2 Hz, 1H, H_5_), 7.92–7.76 (m, 3H, H_2′_, H_2″_, H_6″_), 7.69 (d, *J* = 7.2* Hz*, 1H, H_7_), 7.55 (d, *J* = *8.10 Hz*, 1H, H_6’_), 7.46 (d, *J* = 7.5* Hz*, 1H, H_8_), 7.18 (t, *J* = 7.80 Hz, 1H, H_6_), 7.04–6.95 (m, 1H, H_5′_), 5.00 (s, 2H, CH_2_), 4.00 (s, 3H, OCH_3_) ppm. ^13^C NMR (75 MHz, DMSO-*d*_*6*_): *δ* 166.7, 165.9, 162.7, 162.2, 151.5, 148.7, 148.2, 141.2, 134.3, 132.3, 129.5, 128.2, 128.0, 126.0, 120.8, 115.2, 113.1, 111.0, 67.3, 56.2; Anal. Calcd: C_23_H_18_ClN_3_O_4_; C, 61.88; H, 4.06; N, 12.55; Found; C, 62.07; H, 4.31; N, 12.72.

#### 2-(2-methoxy-4-(4-oxo-3,4-dihydroquinazolin-2-yl)phenoxy)-N-(naphthalen-2-yl)acetamide (7o)

Cream solid; Yield: 65%; MP = 217–219 °C; IR (KBr, v_max_) 3291 (NH), 3065 (CH Aromatic), 2980(CH Aliphatic), 1675 (C=O) Cm^−1^; ^1^H NMR (300 MHz, DMSO-*d*_*6*_) *δ* 12.47 (s, 1H, NH Quinazolinone), 10.10 (s, 1H, NH_Amide_), 8.14 (d, *J* = *7.9 Hz*, 1H, H_5_), 8.08 (d, *J* = *7.9 Hz*, 1H, H_8″_) 7.96 (d, *J* = *7.8 Hz,* 1H, H_3″_), 7.91–7.84 (m, 2H, H_2″_, H_5″_), 7.80–7.75 (m, 3H, H_2′_, H_6′_, H_7_), 7.72 (d, *J* = 8.1 Hz, 1H, H_8_), 7.61–7.43 (m, 4H, H_6_, H_4″_, H_6″_, H_7″_), 7.21 (d, *J* = *8.7 Hz,* 1H, H_5′_), 4.99 (s, 2H, CH_2_), 3.91 (s, 3H, OCH_3_) ppm. ^13^C NMR (75 MHz, DMSO-*d*_*6*_): *δ* 166.9, 162.3, 151.7, 150.0, 148.8, 148.7, 134.4, 133.6, 132.6, 127.4, 125.8, 120.7, 113.2, 111.3, 111.0, 67.8, 59.8; Anal. Calcd: C_27_H_21_N_3_O_4_; C, 71.83; H, 4.69; N, 9.31;Found; C, 71.98; H, 4.87; N, 9.56.

#### 2-(2-methoxy-4-(4-oxo-3,4-dihydroquinazolin-2-yl)phenoxy)-N-(4-methylbenzyl)acetamide (7p)

Cream solid; Yield: 87%; MP = 191–193 °C; IR (KBr, v_max_) 3258 (NH), 3030(CH Aromatic), 2910 (CHAliphatic), 1666(C=O)Cm^−1^; ^1^H NMR(300 MHz, DMSO-*d*_*6*_) *δ* 12.44 (s, 1H, NH Quinazolinone), 8.47 (t, *J* = *6.10 Hz*, 1H, NH_Amide_), 8.13 (d, *J* = *7.80 Hz*, 1H, H_5_), 7.83 (s, 1H, H_2′_) 7.82–7.75 (m, 2H, H_6′_, H_7_), 7.71 (d, *J* = *8.00 Hz*, 1H, H_8_), 7.48 (t, *J* = 7.40 Hz, 1H, H_6_), 7.16–7.06 (m, 4H, H _Phenyl_), 7.05 (d, *J* = *8.10 Hz*, 1H, H_5′_), 4.65 (s, 2H, CH_2_), 4.30 (d, *J* = 5.90 Hz, 2H, CH_2_), 3.89 (s, 3H, OCH_3_), 2.26 (s, 3H, CH_3_) ppm. ^13^C NMR (75 MHz, DMSO-*d*_*6*_): *δ* 167.3, 162.3, 151.7, 150.0, 148.8, 136.0, 135.8, 134.5, 128.8, 127.2, 126.0, 125.8, 121.0, 120.7, 113.3, 11.3, 67.9, 55.4, 41.6, 20.6; Anal. Calcd: C_25_H_23_N_3_O_4_; C, 69.92; H, 5.40; N, 9.78; Found; C, 70.13; H, 5.69; N, 9.97.

#### N-(4-fluorobenzyl)-2-(2-methoxy-4-(4-oxo-3,4-dihydroquinazolin-2-yl)phenoxy)acetamide (7q)

Cream solid; Yield: 76%; MP = 224–226 °C; IR (KBr, v_max_) 3314 (NH), 3050(CH Aromatic), 2960 (CHAliphatic),1671(C=O) Cm^−1^; ^1^H NMR(300 MHz,DMSO-*d*_6_) *δ* 12.44 (s, 1H, NH Quinazolinone), 8.55 (t, *J* = *6.10 Hz*, 1H, NH_Amide_), 8.13 (d, *J* = *7.90 Hz*, 1H, H_5_), 7.89–7.75 (m, 3H, H_2′_, H_6′_, H_7_) 7.70 (d, *J* = *7.8 Hz,* 1H, H_8_), 7.46 (t, *J* = *7.4 Hz*, 1H, H_6_), 7.34–7.27 (m, *2*H, H_2″_, H_6″_), 7.18–7.08 (m, 2H, H_3″_, H_5″_), 7.05 (d, *J* = *8.10 Hz*, 1H, H_5′_), 4.66 (s, 2H, CH_2_), 4.34 (d, *J* = 5.90 Hz, 2H, CH_2 Benzyl_), 3.89 (s, 3H, OCH_3_) ppm. ^13^C NMR (75 MHz, DMSO-*d*_*6*_): *δ* 167.5, 162.7, 160.9(d, ^1^*J*_CF_ = 208.5), 151.6, 149.9, 148.8, 135.3, 129.2, 126.0, 125.8, 121.0, 120.7, 115.0, 113.3, 111.2, 110.9, 67.8, 55.9, 41.2; Anal. Calcd: C_24_H_20_FN_3_O_4_; C, 66.51; H, 4.65; N, 9.69; Found; C, 66.72; H, 4.84; N, 9.91.

#### N-benzyl-2-(2-methoxy-4-(4-oxo-3,4-dihydroquinazolin-2-yl)phenoxy)acetamide (7r)

Cream solid; Yield: 81%; MP = 194–196 °C; IR (KBr, v_max_) 3264 (NH), 3025(CH Aromatic), 2960 (CH Aliphatic),1662 (C=O) Cm^−1^; ^1^H NMR (300 MHz,DMSO-*d*_*6*_) *δ* 12.45 (s, 1H, NH Quinazolinone), 8.53 (t, *J* = *6.10 Hz*, 1H, NH_Amide_), 8.13 (d, *J* = *7.90 Hz*, 1H, H_5_), 7.84 (s, 1H, H_2′_) 7.83–7.75 (m, 2H, H_6′_, H_7_), 7.71 (d, *J* = *8.10 Hz*, 1H, H_8_), 7.48 (t, *J* = 7.50 Hz, 1H, H_6_), 7.35–7.20 (m, 5H, H _Phenyl_), 7.06 (d, *J* = *8.10 Hz*, 1H, H_5′_), 4.67 (s, 2H, CH_2_), 4.35 (d, *J* = 6.00 Hz, 2H, CH_2_), 3.89 (s, 3H, OCH_3_) ppm. ^13^C NMR (75 MHz, DMSO-*d*_*6*_): *δ* 167.4, 162.3, 151.7, 150.0, 148.8, 139.1, 134.6, 128.2, 127.2, 126.8, 126.8, 126.19, 15.8, 120.7, 113.4, 111.0, 67.9, 55.4, 41.9; Anal. Calcd: C_24_H_21_N_3_O_4_; C, 69.39; H, 5.10; N, 10.11; Found; C, 69.57; H, 5.28; N, 10.26.

### α-glucosidase inhibition assay

The anti-α-glucosidase effects of synthesized compounds, **7a–r** were screened according to the previously reported method [7, 12, 25].

### Enzyme kinetic studies

The mode of inhibition of the most active compound **7b**, identified with the lowest IC_50_, was investigated against an α-glucosidase activity with different concentrations of *p*-nitrophenyl *α*-d-glucopyranoside (1–10 mM) as substrate in the absence and presence of **7b** at different concentrations (0, 3.6, 7.2, and 14.4 µM). A Lineweaver–Burk plot was generated to identify the type of inhibition and the Michaelis–Menten constant (*K*_m_) value was determined from the plot between the reciprocal of the substrate concentration (1/[S]) and reciprocal of enzyme rate (1/V) over various inhibitor concentrations. The experimental inhibitor constant (*K*_i_) value was constructed by secondary plots of the inhibitor concentration [I] versus *K*_m_ [3, 7].

### Molecular docking

The molecular docking studies were performed using the Maestro Molecular Modeling platform (version 10.5) by Schrödinger, LLC. The X-ray crystal structure of the receptor (PDB ID: 5NN8) was extracted from the PDB database. The protein is then prepared using a protein preparation wizard so that co-crystallized ligands and all water molecules were removed, the missing side chains and loops were filled using the prime tool, and PROPKA assigned H-bonds at pH: 7.4. To prepare the ligands, the 2D structures of the ligands were drawn in ChemDraw and converted into SDF files and subjected to ligprep module. Ligands were prepared by OPLS_2005 force field using EPIK. The grid box was generated for each binding site using entries with a box size of 25 A, the derivative was docked on binding sites using induced-fit docking, reporting 10 poses per ligand to form the final complex [14, 26].

## Supplementary Information

**Additional file 1**: **Figure S1.** 2-(2-methoxy-4-(4-oxo-3,4-dihydroquinazolin-2-yl)phenoxy)-N-phenylacetamide (**7a**). **Figure S2.** N-(2-fluorophenyl)-2-(2-methoxy-4-(4-oxo-3,4-dihydroquinazolin-2-yl)phenoxy)acetamide (**7b**). **Figure S3.** N-(4-fluorophenyl)-2-(2-methoxy-4-(4-oxo-3,4-dihydroquinazolin-2-yl)phenoxy)acetamide (**7c**). **Figure S4.** N-(2-chlorophenyl)-2-(2-methoxy-4-(4-oxo-3,4-dihydroquinazolin-2-yl)phenoxy)acetamide (**7d**). **Figure S5.** N-(3-chlorophenyl)-2-(2-methoxy-4-(4-oxo-3,4-dihydroquinazolin-2-yl)phenoxy)acetamide (**7e**). **Figure S6.** N-(4-chlorophenyl)-2-(2-methoxy-4-(4-oxo-3,4-dihydroquinazolin-2-yl)phenoxy)acetamide (**7f**). **Figure S7.** N-(4-bromophenyl)-2-(2-methoxy-4-(4-oxo-3,4-dihydroquinazolin-2-yl)phenoxy)acetamide (**7g**). **Figure S8.** 2-(2-methoxy-4-(4-oxo-3,4-dihydroquinazolin-2-yl)phenoxy)-N-(o-tolyl)acetamide (**7 h**). **Figure S9.** 2-(2-methoxy-4-(4-oxo-3,4-dihydroquinazolin-2-yl)phenoxy)-N-(p-tolyl)acetamide (**7i**). **Figure S10.** N-(2,6-dimethylphenyl)-2-(2-methoxy-4-(4-oxo-3,4-dihydroquinazolin-2-yl)phenoxy)acetamide (**7j**). **Figure S11.** N-(4-ethylphenyl)-2-(2-methoxy-4-(4-oxo-3,4-dihydroquinazolin-2-yl)phenoxy)acetamide (**7k**). **Figure S12.** 2-(2-methoxy-4-(4-oxo-3,4-dihydroquinazolin-2-yl)phenoxy)-N-(4-methoxyphenyl)acetamide (**7 l**). **Figure S13.** N-(4-hydroxyphenyl)-2-(2-methoxy-4-(4-oxo-3,4-dihydroquinazolin-2-yl)phenoxy)acetamide (**7m**). **Figure S14.** 2-(2-methoxy-4-(4-oxo-3,4-dihydroquinazolin-2-yl)phenoxy)-N-(4-nitrophenyl)acetamide (**7n**). **Figure S15.** 2-(2-methoxy-4-(4-oxo-3,4-dihydroquinazolin-2-yl)phenoxy)-N-(naphthalen-2-yl)acetamide (**7o**). **Figure S16.** 2-(2-methoxy-4-(4-oxo-3,4-dihydroquinazolin-2-yl)phenoxy)-N-(4-methylbenzyl)acetamide (**7p**). **Figure S17.** N-(4-fluorobenzyl)-2-(2-methoxy-4-(4-oxo-3,4-dihydroquinazolin-2-yl)phenoxy)acetamide (**7q**). **Figure S18.** N-benzyl-2-(2-methoxy-4-(4-oxo-3,4-dihydroquinazolin-2-yl)phenoxy)acetamide (**7r**).

## Data Availability

The datasets generated and/or analysed during the current study are available in the Worldwide Protein Data Bank (wwPDB) repository. (http://www.rcsb.org).
