# Supplementary material for: Synthesis and bioactivities evaluation of quinazolin-4(3H)-one derivatives as α-glucosidase inhibitors
Source: BMC Chem. 2022 Nov 15;16(1):97. doi: 10.1186/s13065-022-00885-z (PMC9667588; doi:10.1186/s13065-022-00885-z)

**Additional file 1**

*Fig. S1. 2-(2-methoxy-4-(4-oxo-3,4-dihydroquinazolin-2-yl)phenoxy)-N-phenylacetamide (****7a****)*


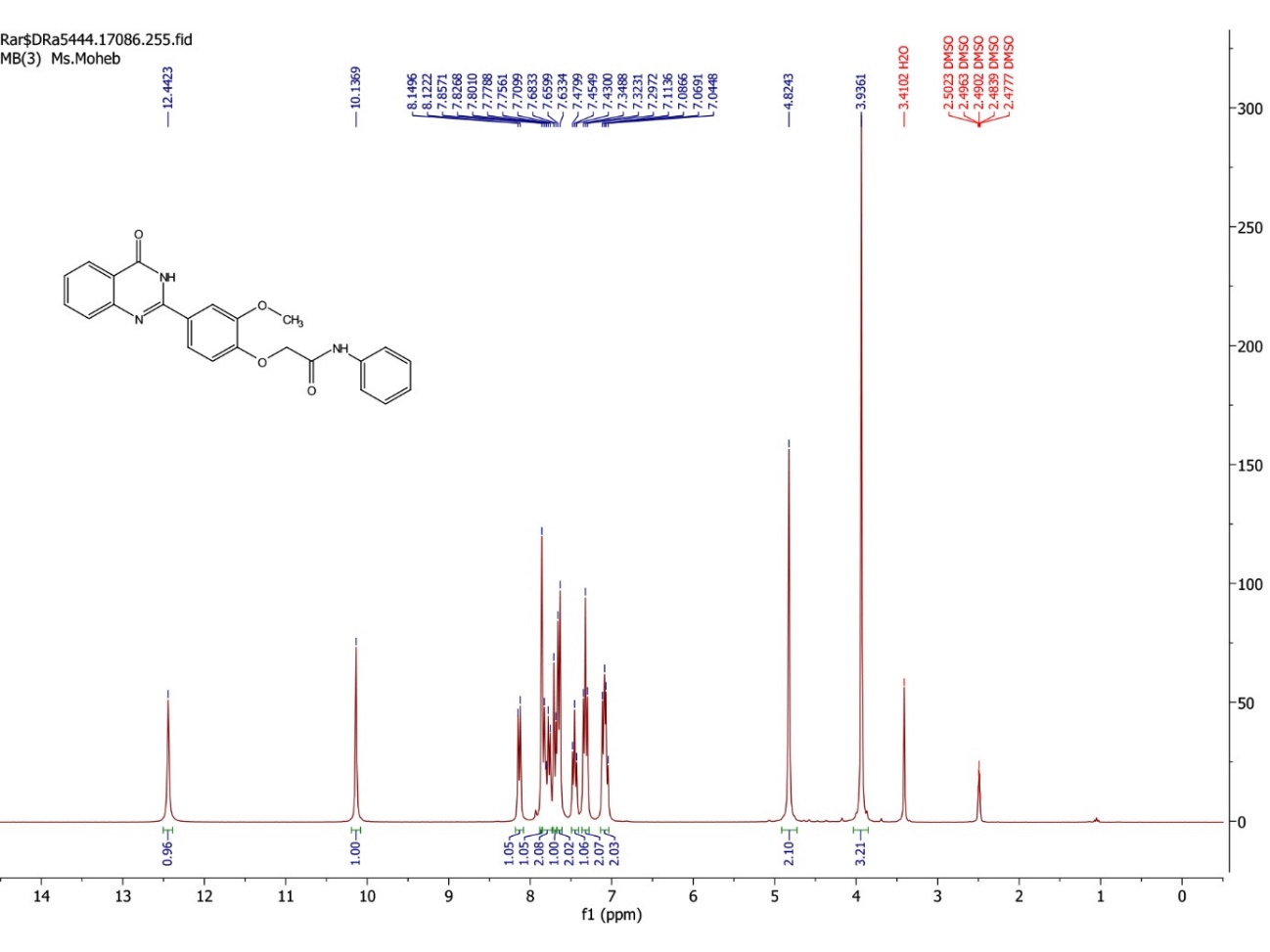


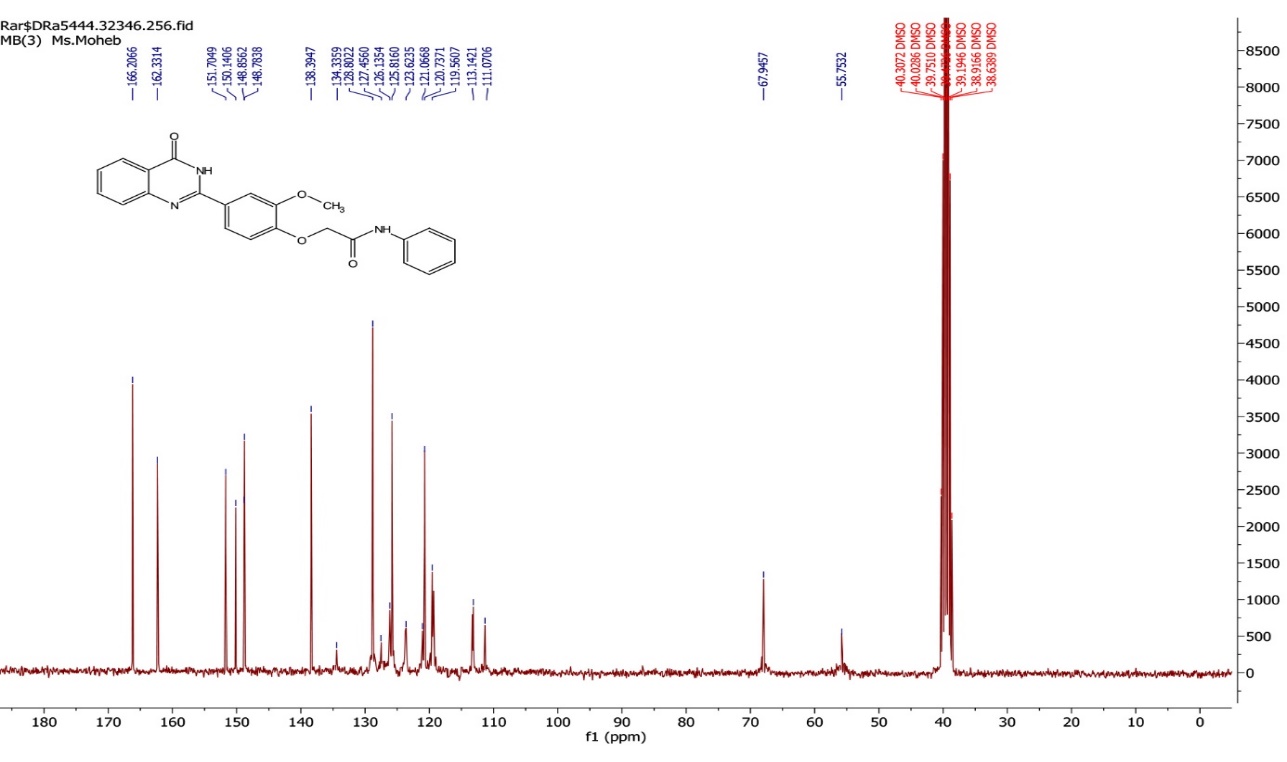


*Fig. S2.N-(2-fluorophenyl)-2-(2-methoxy-4-(4-oxo-3,4-dihydroquinazolin-2-yl)phenoxy)acetamide (****7b****)*


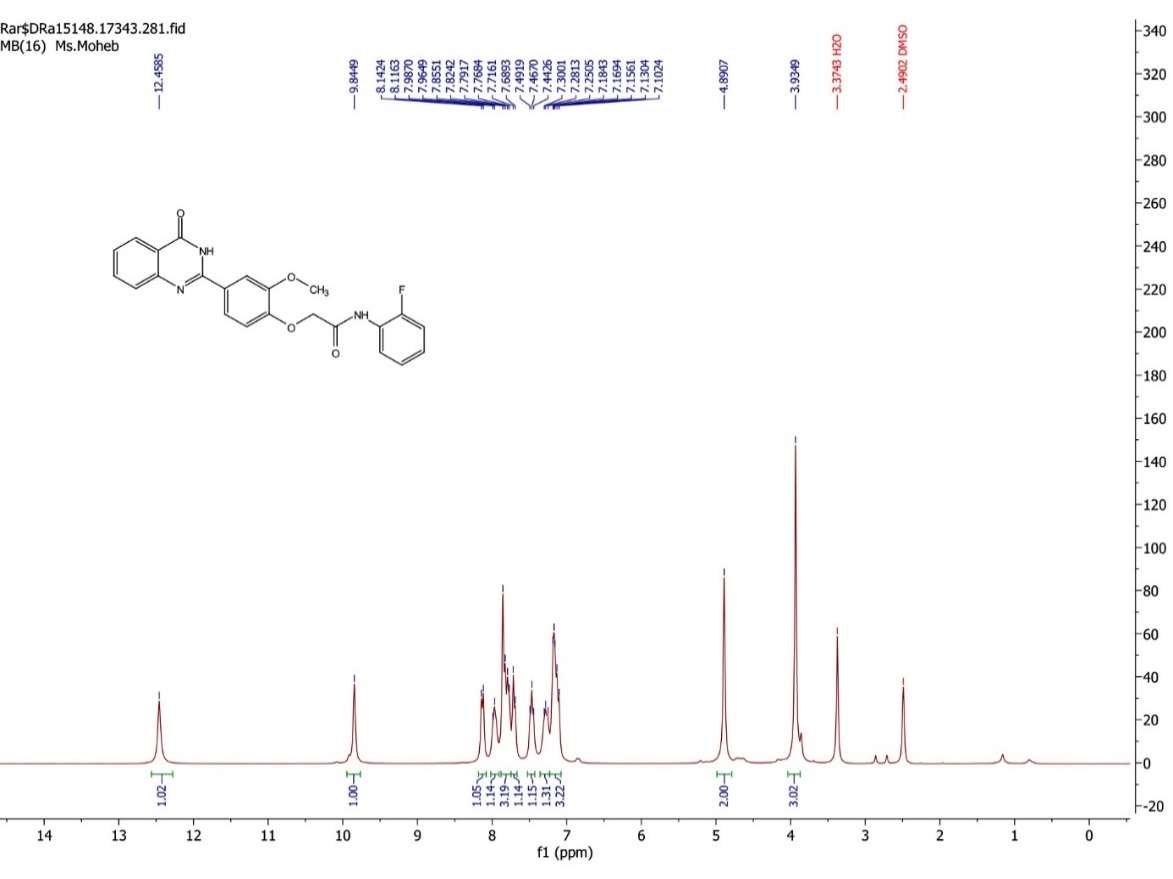


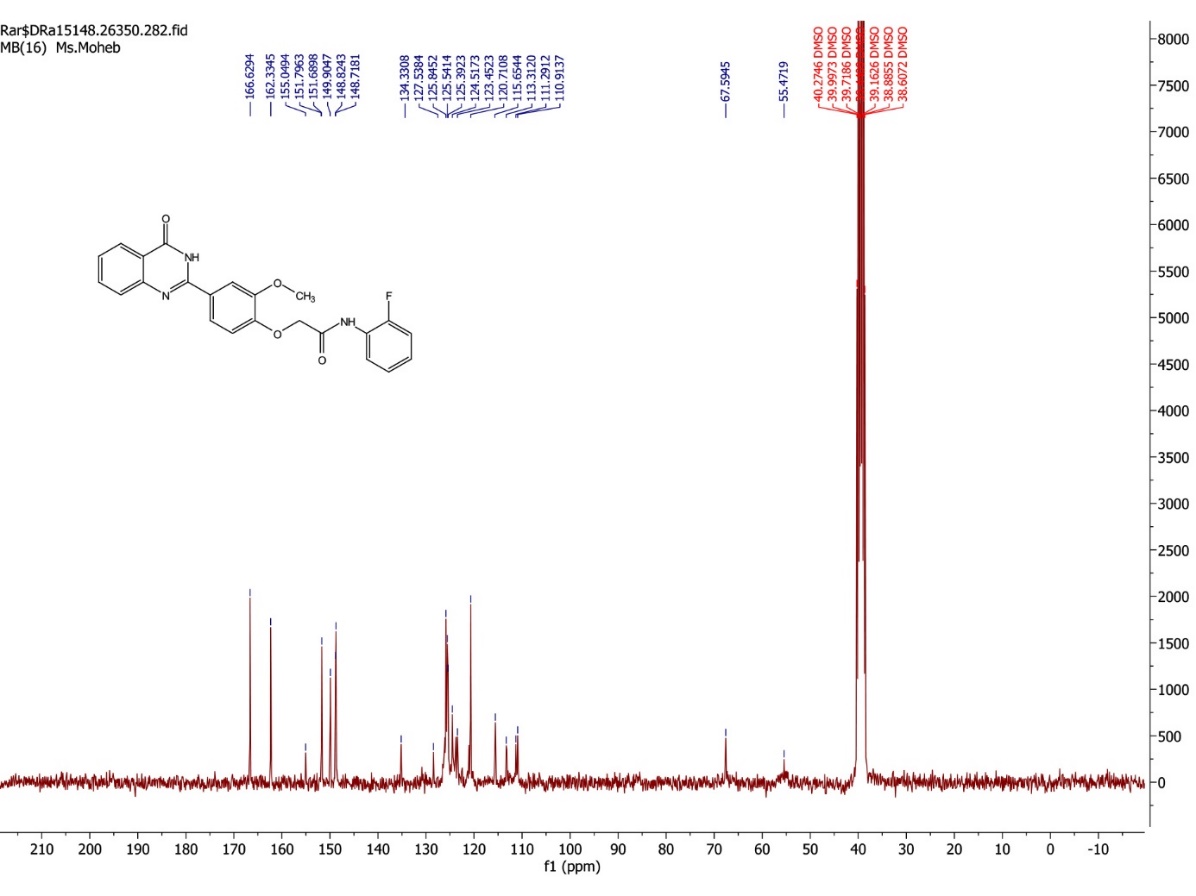


*Fig. S3.N-(4-fluorophenyl)-2-(2-methoxy-4-(4-oxo-3,4-dihydroquinazolin-2-yl)phenoxy)acetamide (****7c****)*


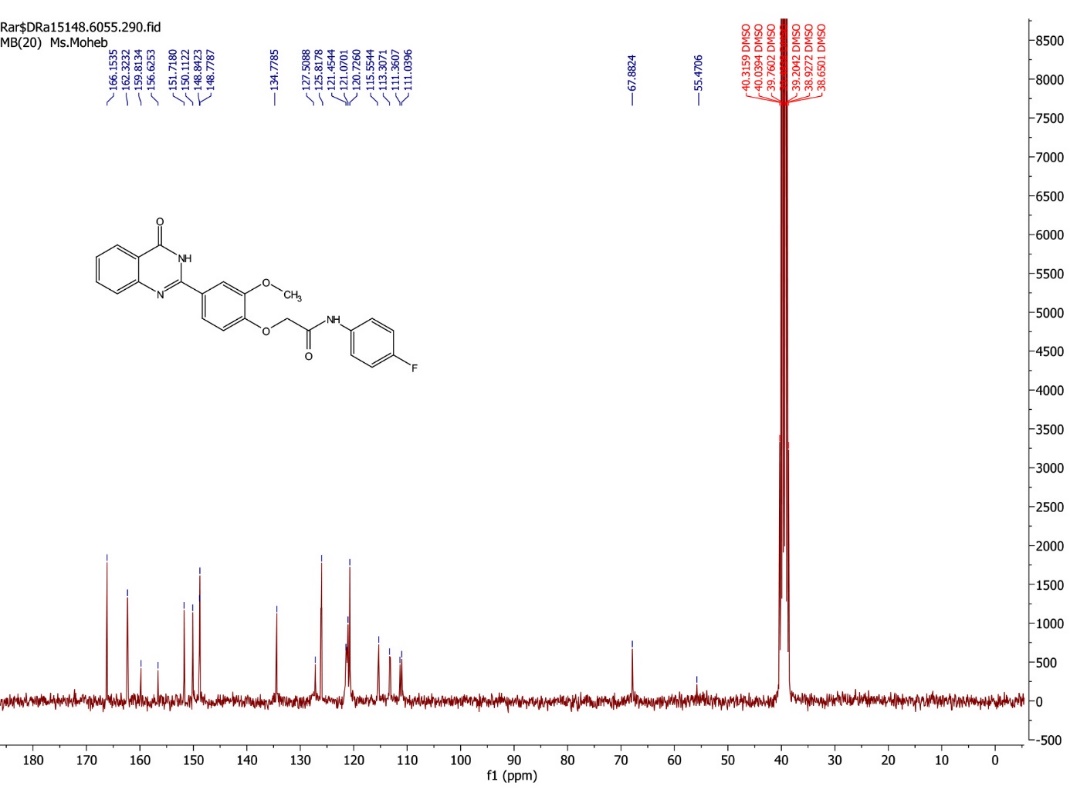


*Fig. S4.N-(2-chlorophenyl)-2-(2-methoxy-4-(4-oxo-3,4-dihydroquinazolin-2-yl)phenoxy)acetamide (****7d****)*


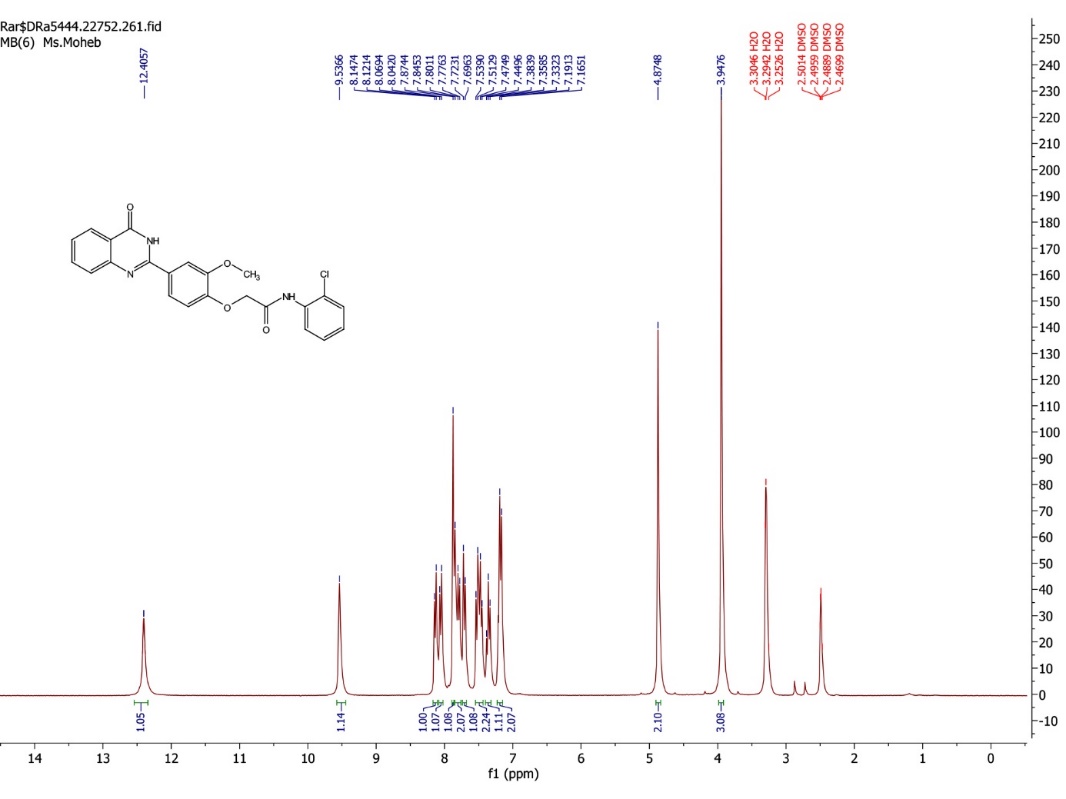


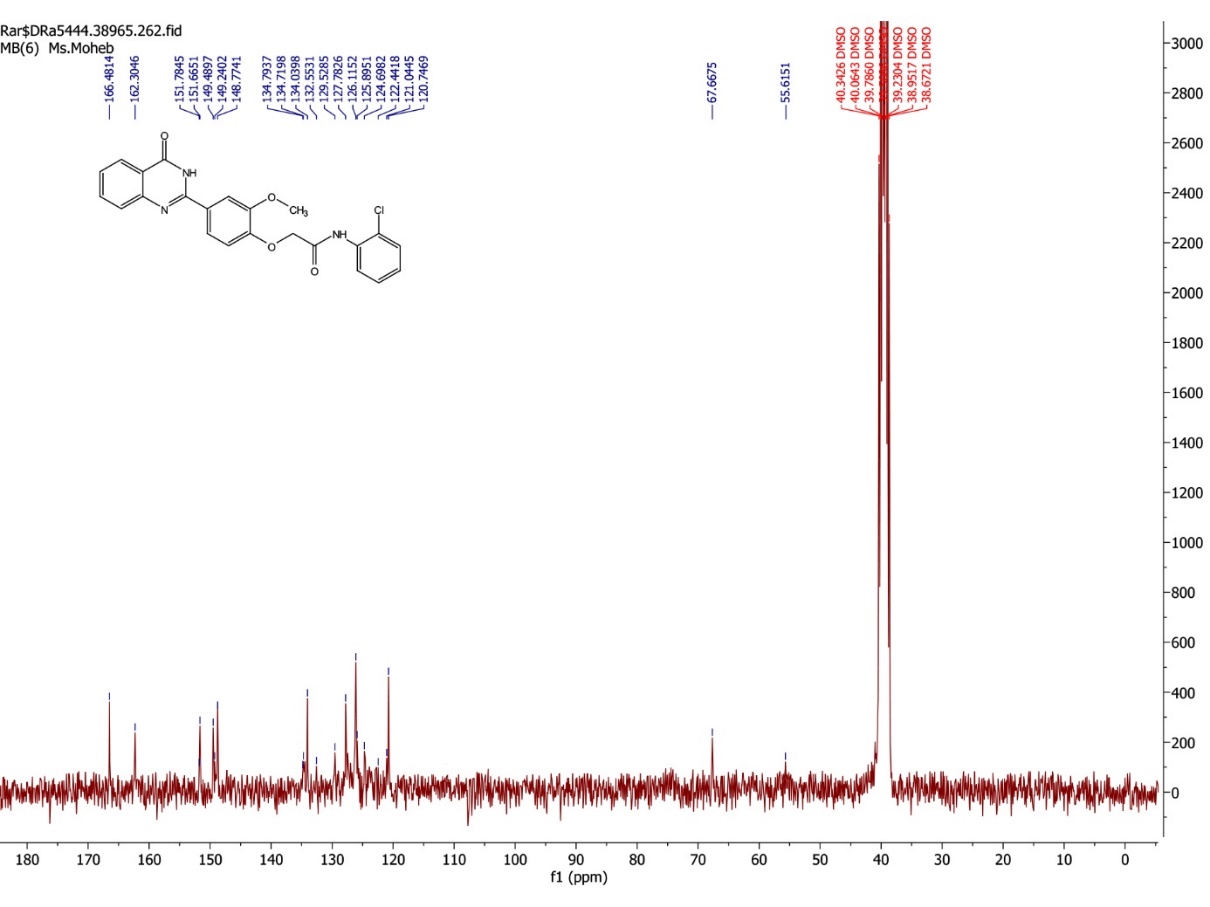


*Fig. S5.N-(3-chlorophenyl)-2-(2-methoxy-4-(4-oxo-3,4-dihydroquinazolin-2-yl)phenoxy)acetamide (****7e****)*


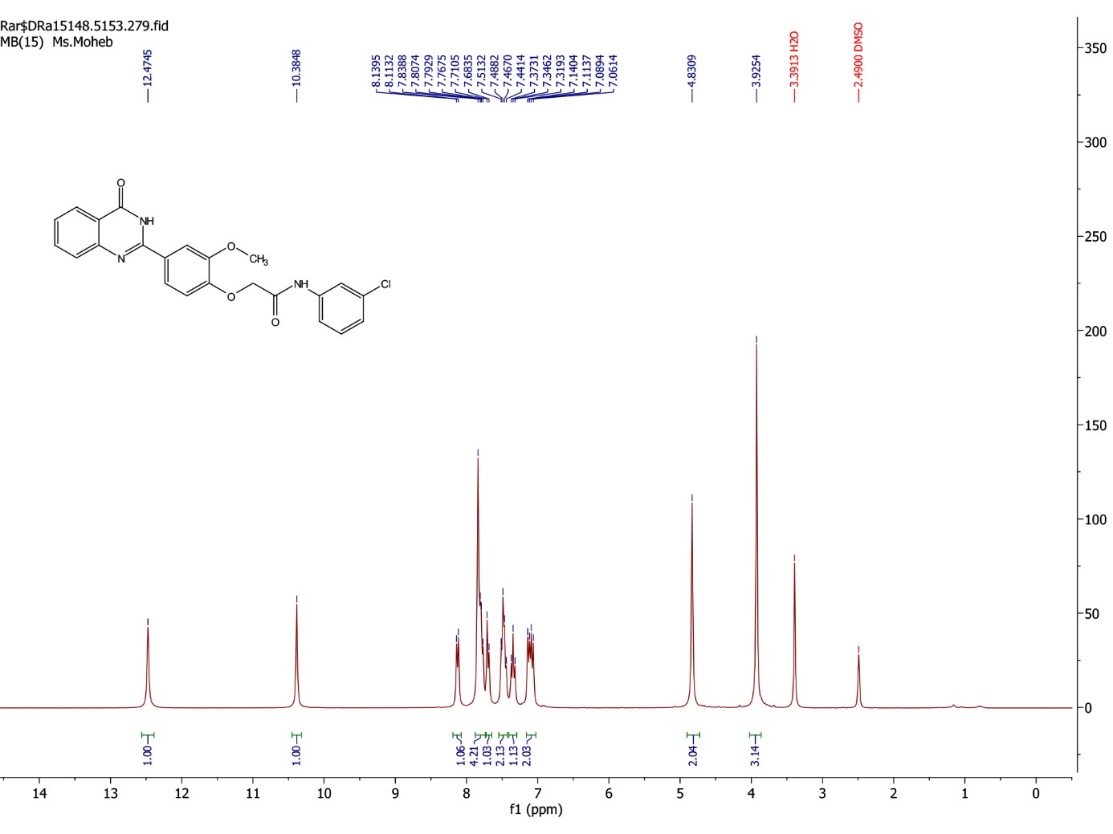


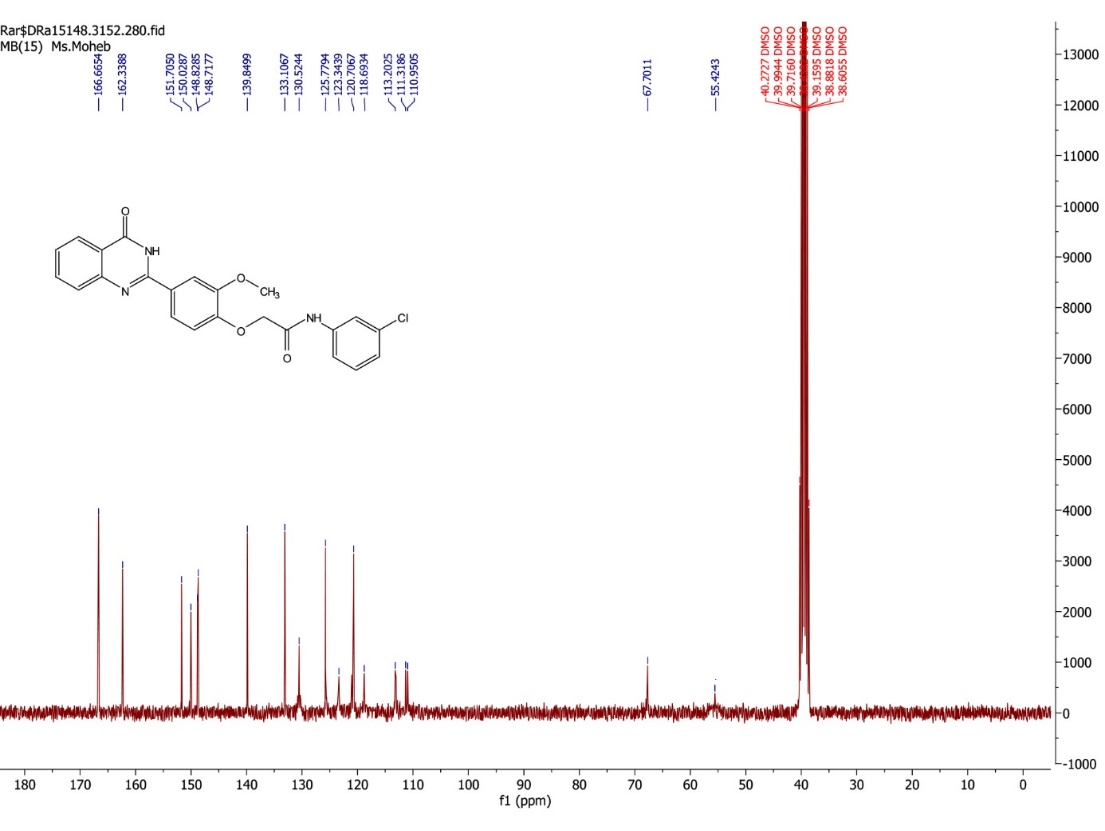


*Fig. S6.N-(4-chlorophenyl)-2-(2-methoxy-4-(4-oxo-3,4-dihydroquinazolin-2-yl)phenoxy)acetamide (****7f****)*


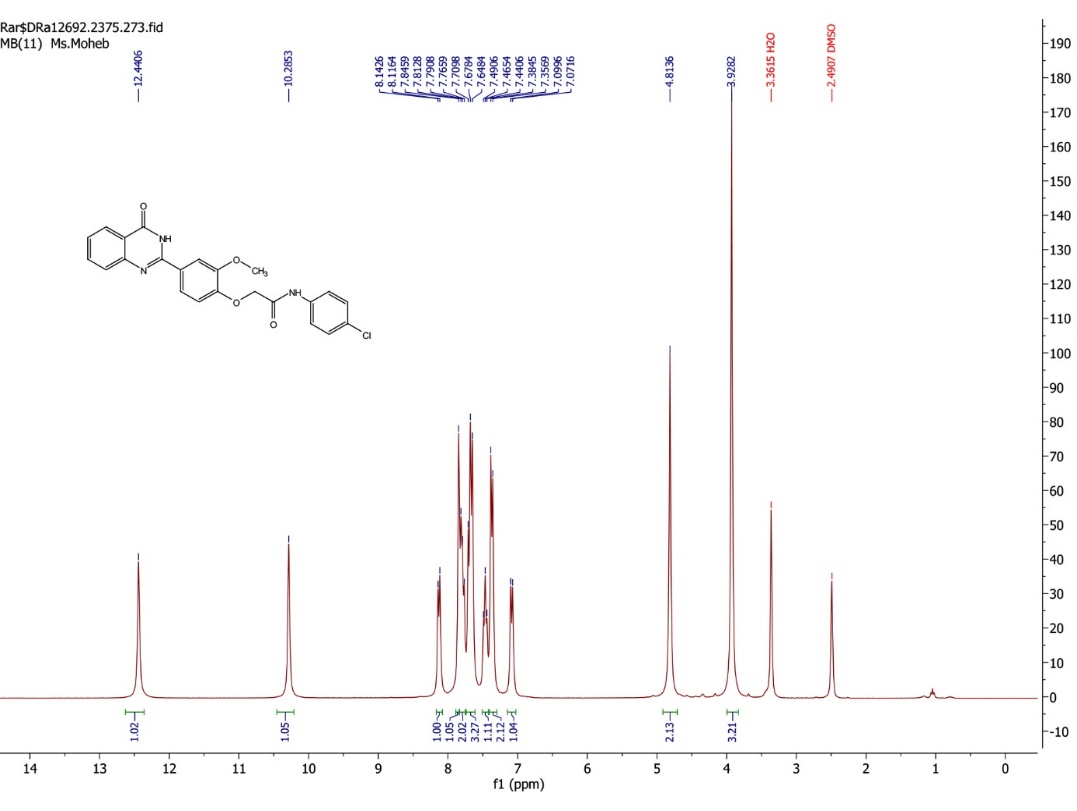


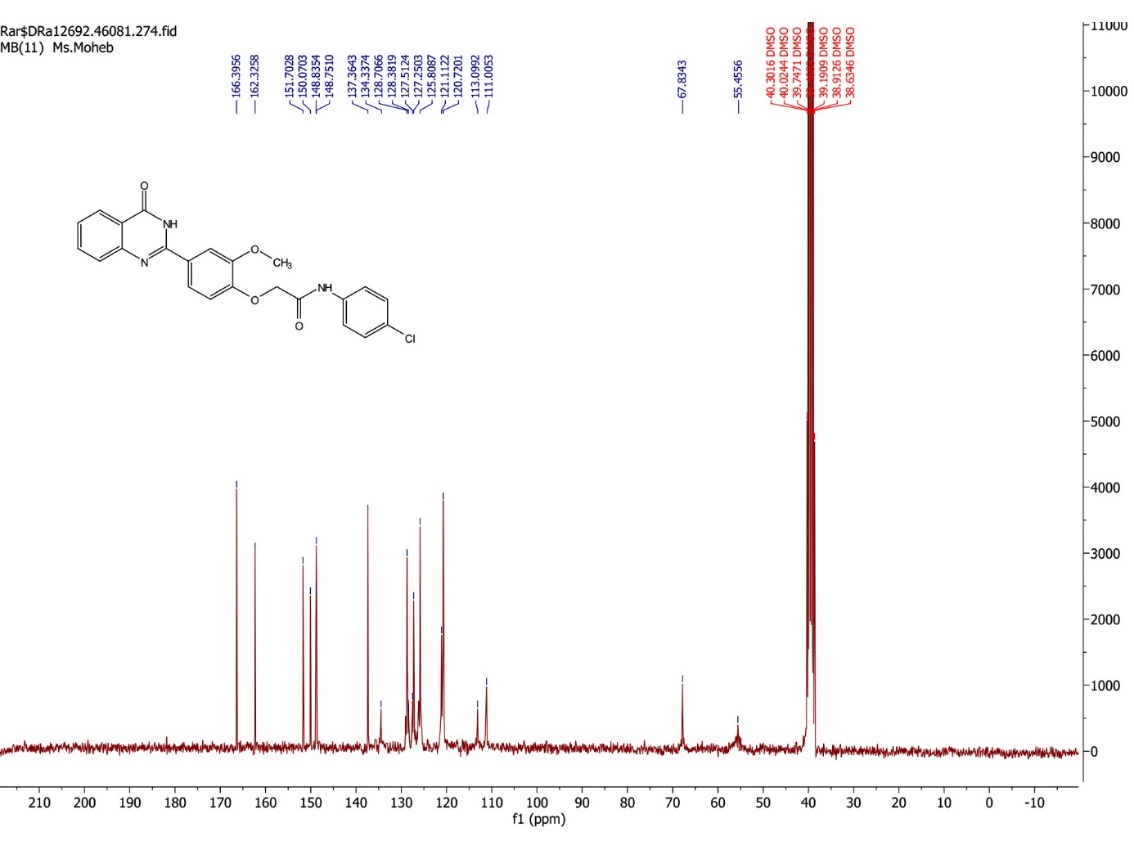


*Fig. S7.N-(4-bromophenyl)-2-(2-methoxy-4-(4-oxo-3,4-dihydroquinazolin-2-yl)phenoxy)acetamide (****7g****)*


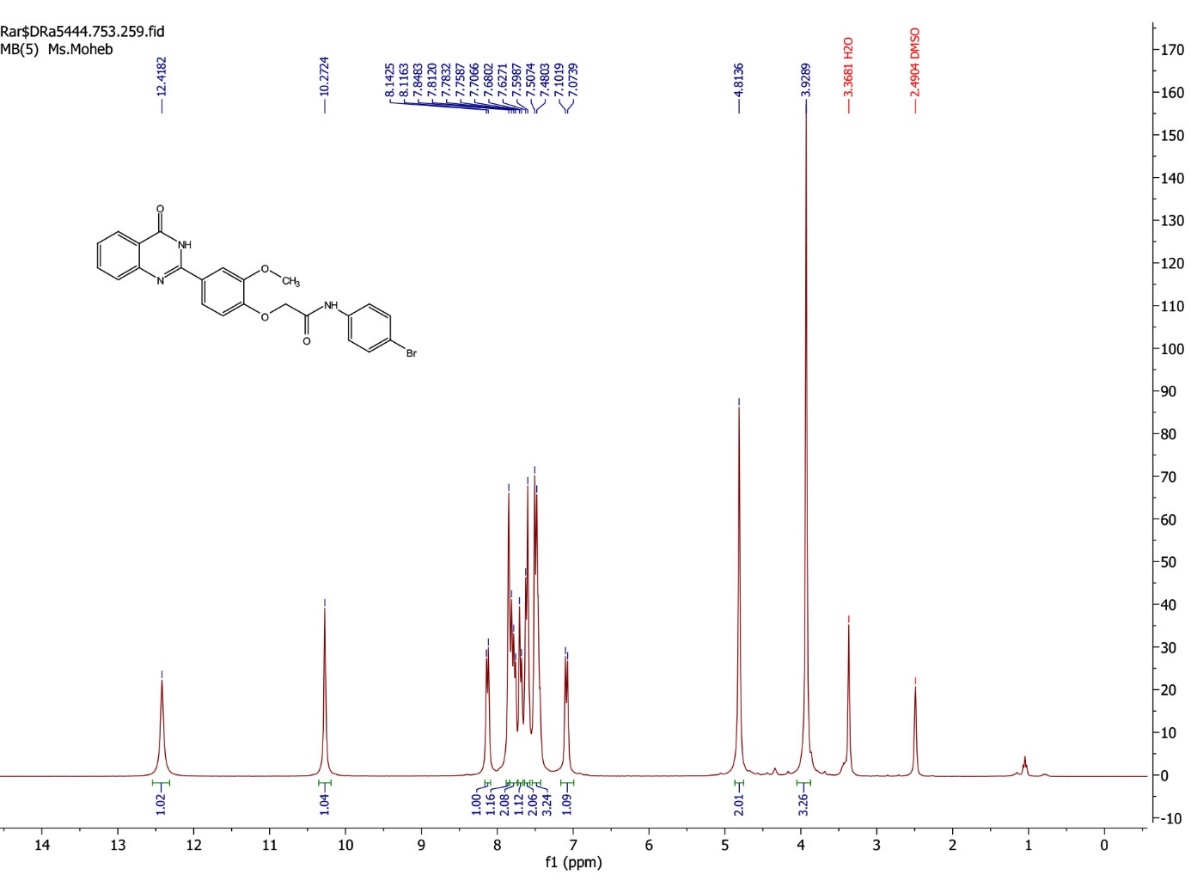


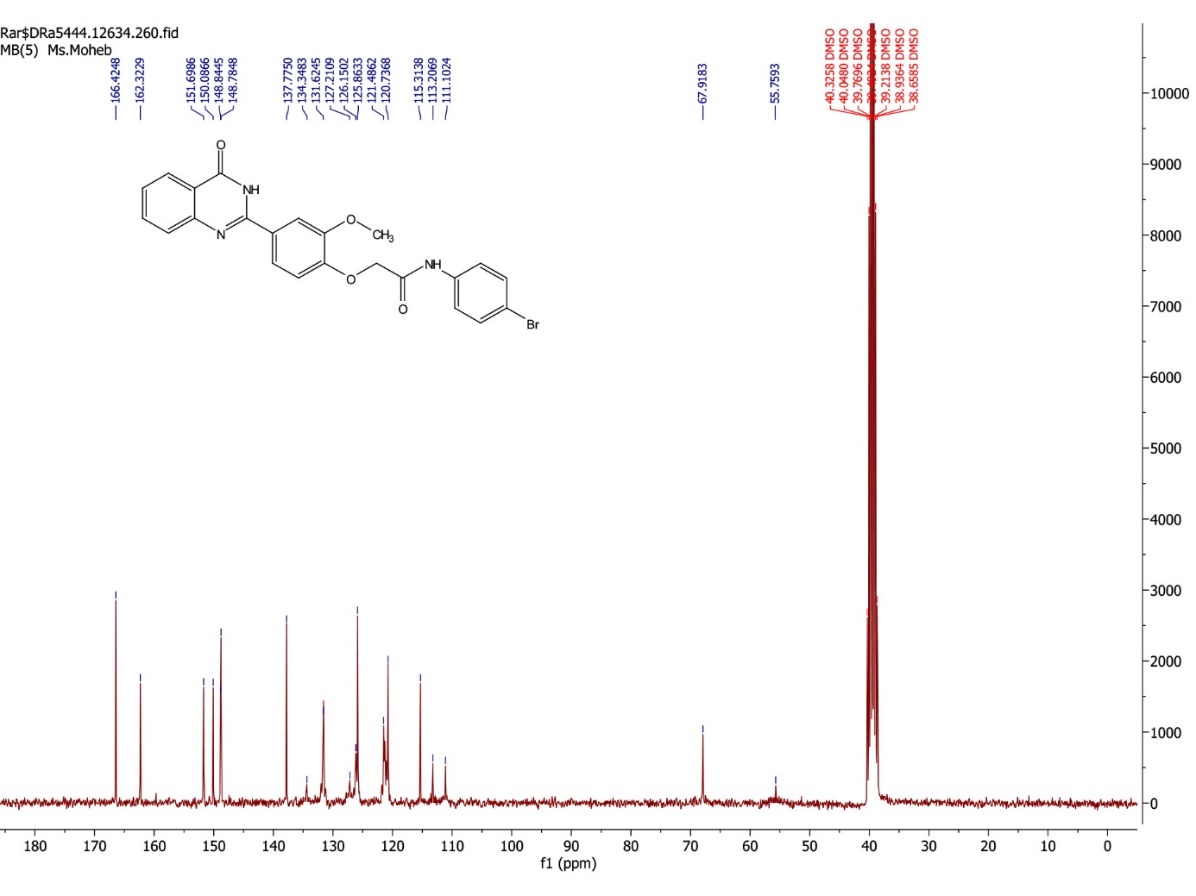


*Fig. S8.2-(2-methoxy-4-(4-oxo-3,4-dihydroquinazolin-2-yl)phenoxy)-N-(o-tolyl)acetamide (****7h****)*


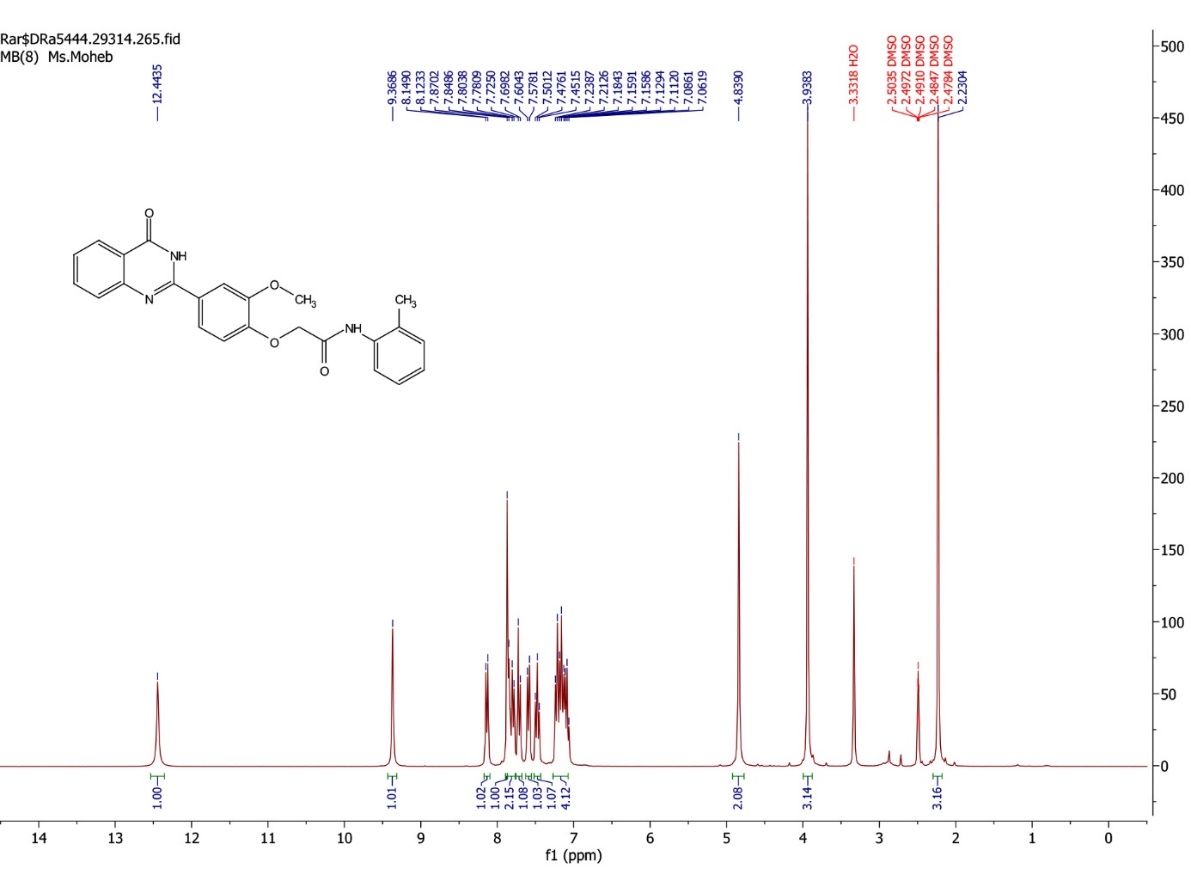


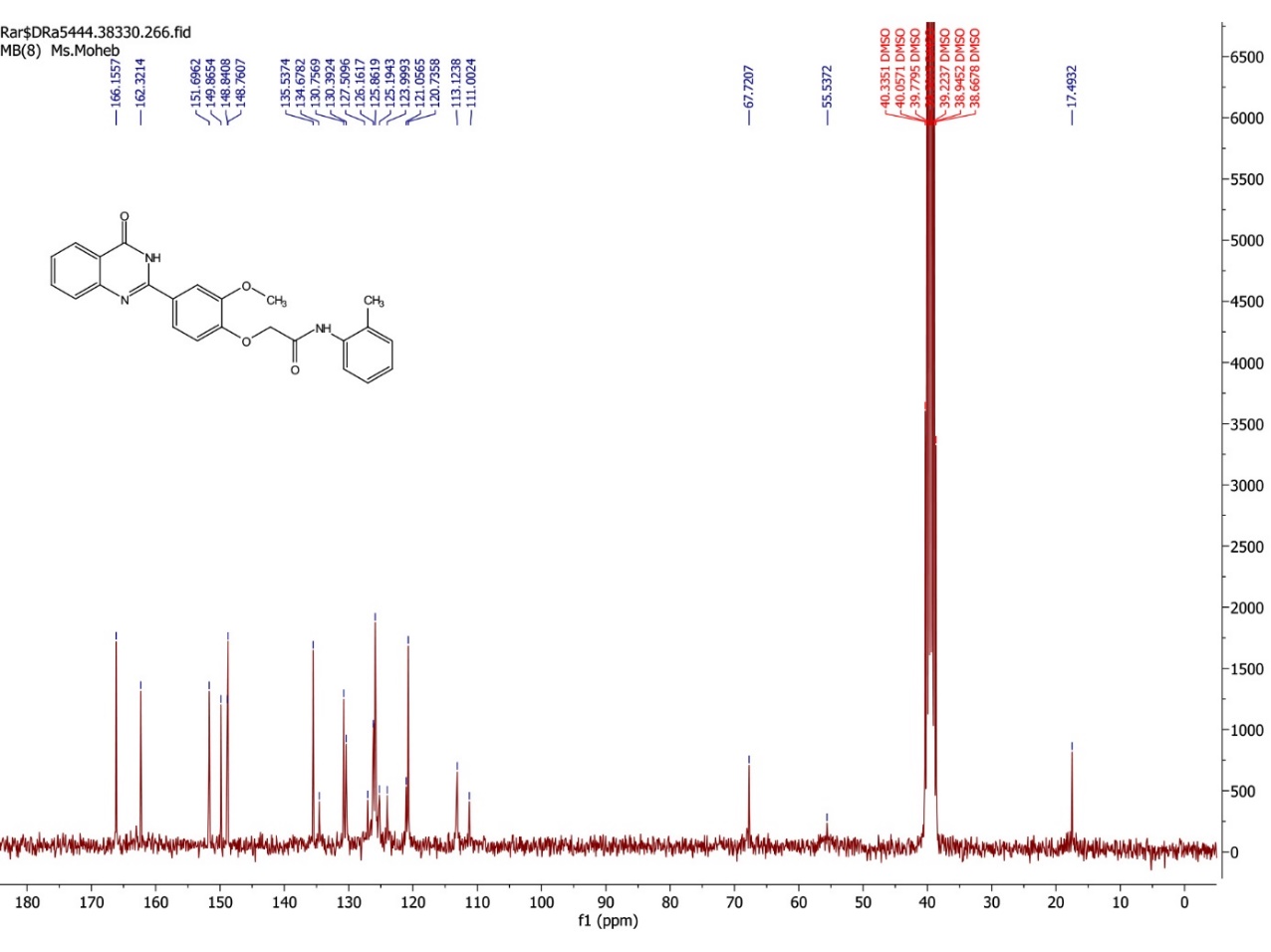


*Fig. S9.2-(2-methoxy-4-(4-oxo-3,4-dihydroquinazolin-2-yl)phenoxy)-N-(p-tolyl)acetamide (***7i**)


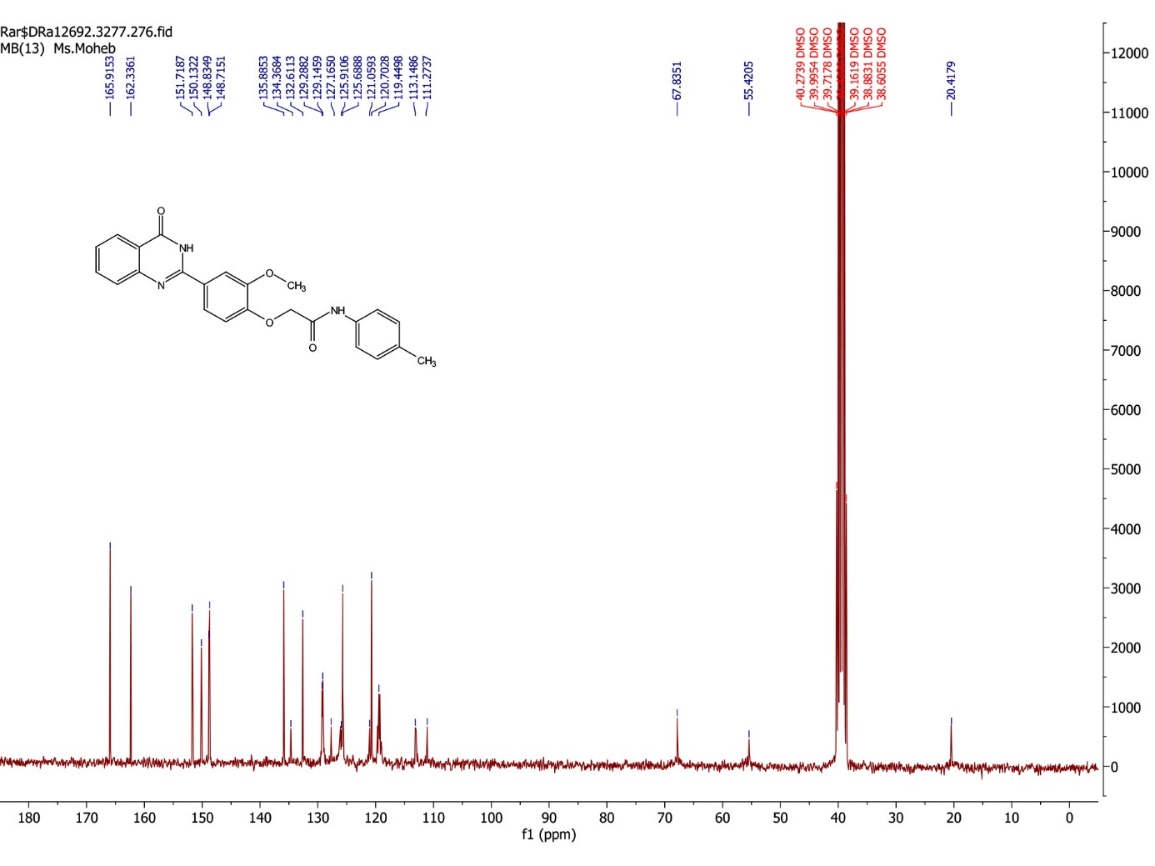


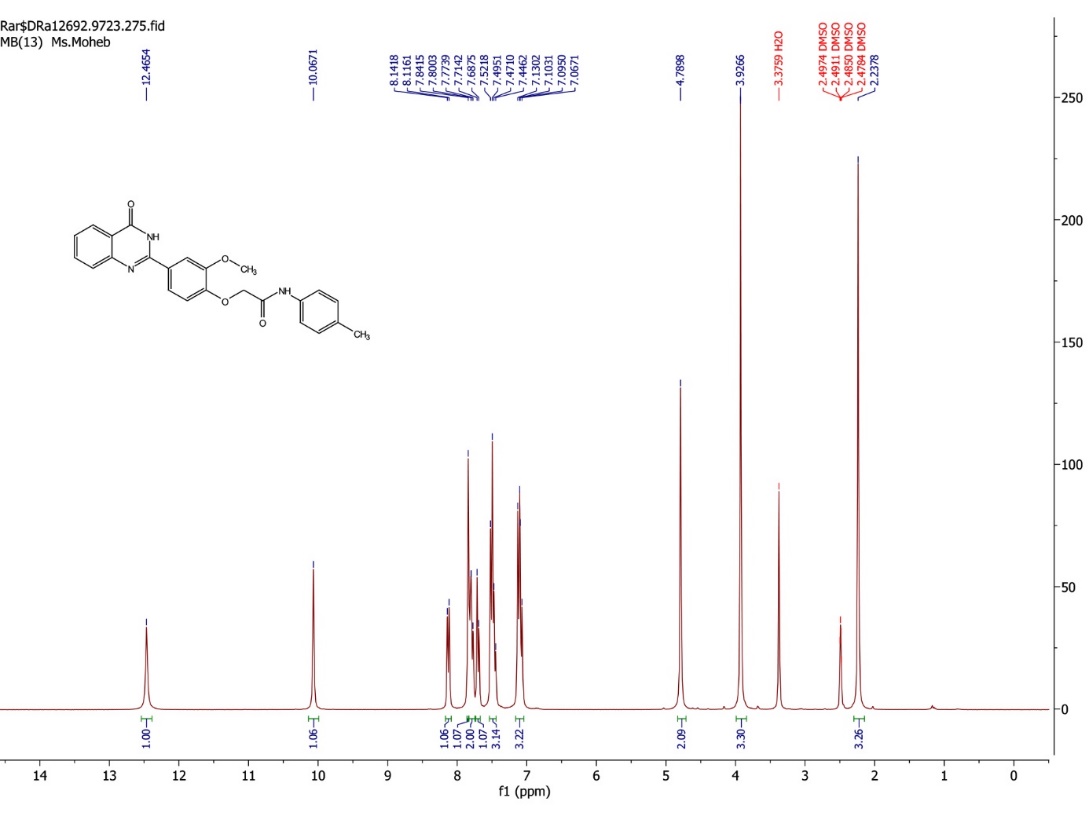


*Fig. S10.N-(2,6-dimethylphenyl)-2-(2-methoxy-4-(4-oxo-3,4-dihydroquinazolin-2-yl)phenoxy)acetamide (****7j****)*
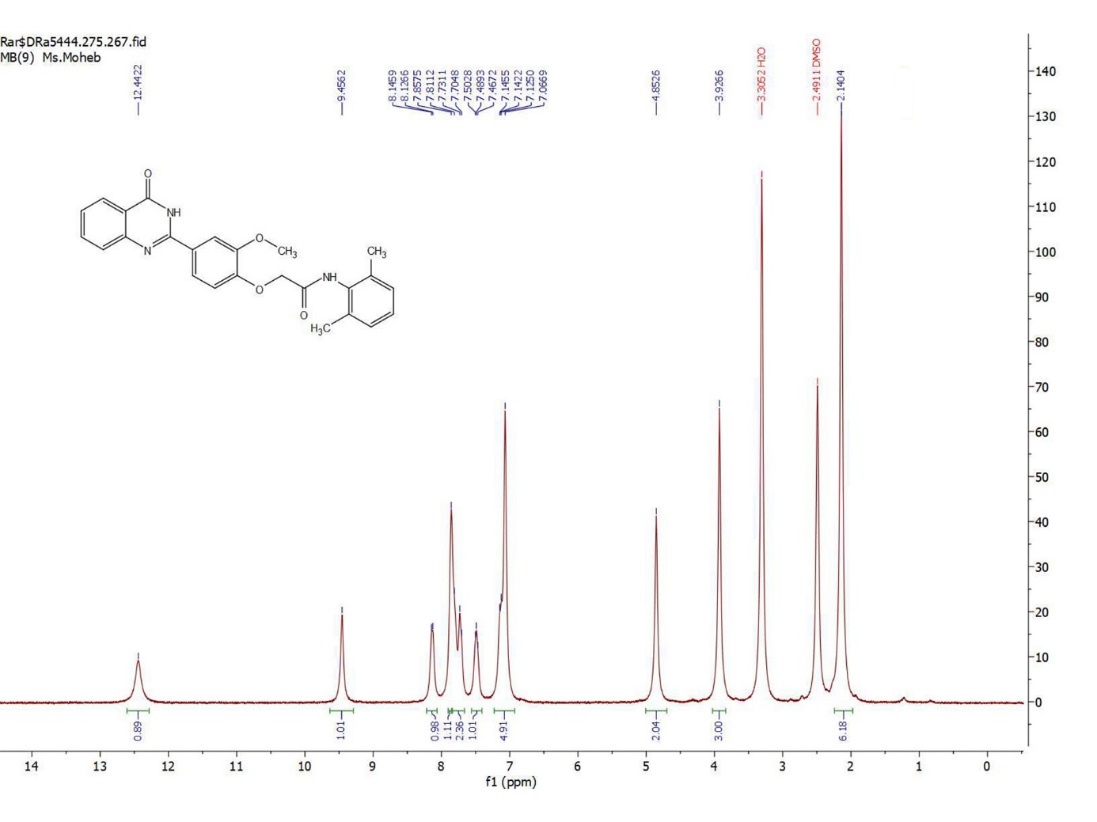


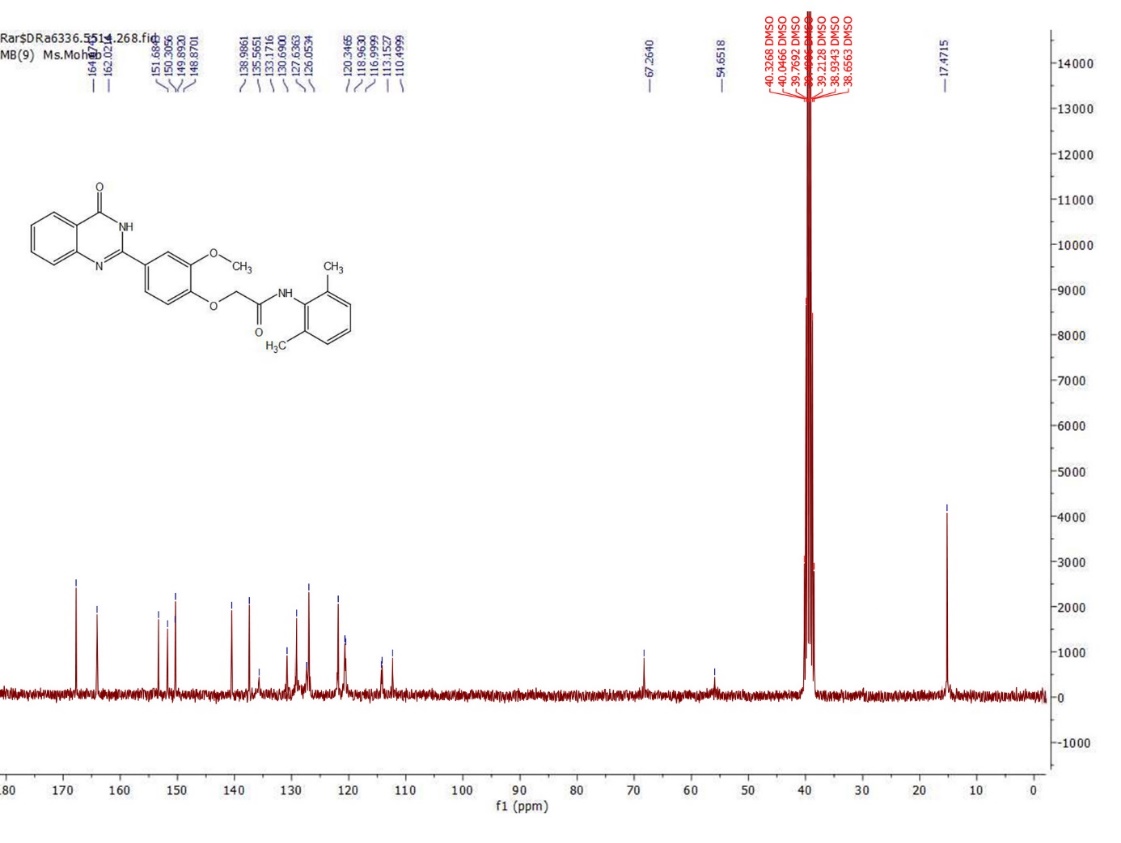


*Fig. S11.N-(4-ethylphenyl)-2-(2-methoxy-4-(4-oxo-3,4-dihydroquinazolin-2-yl)phenoxy)acetamide (****7k****)*


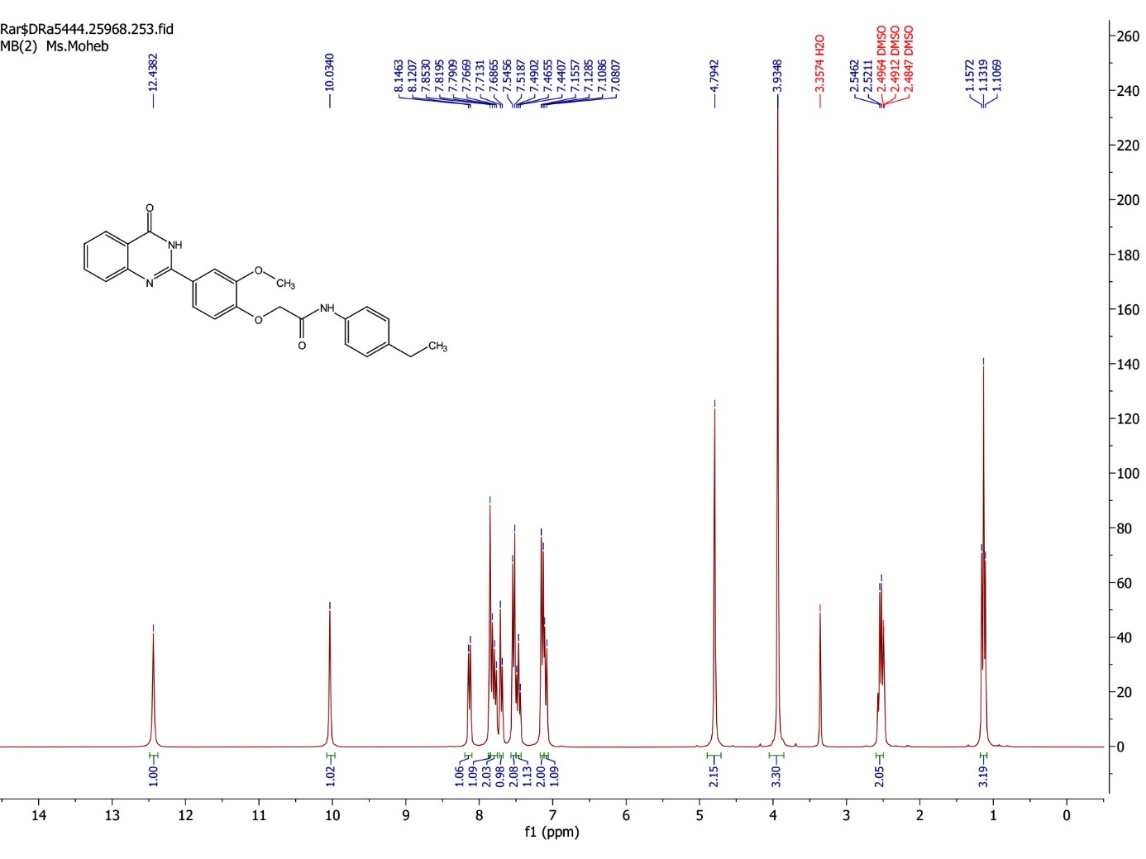


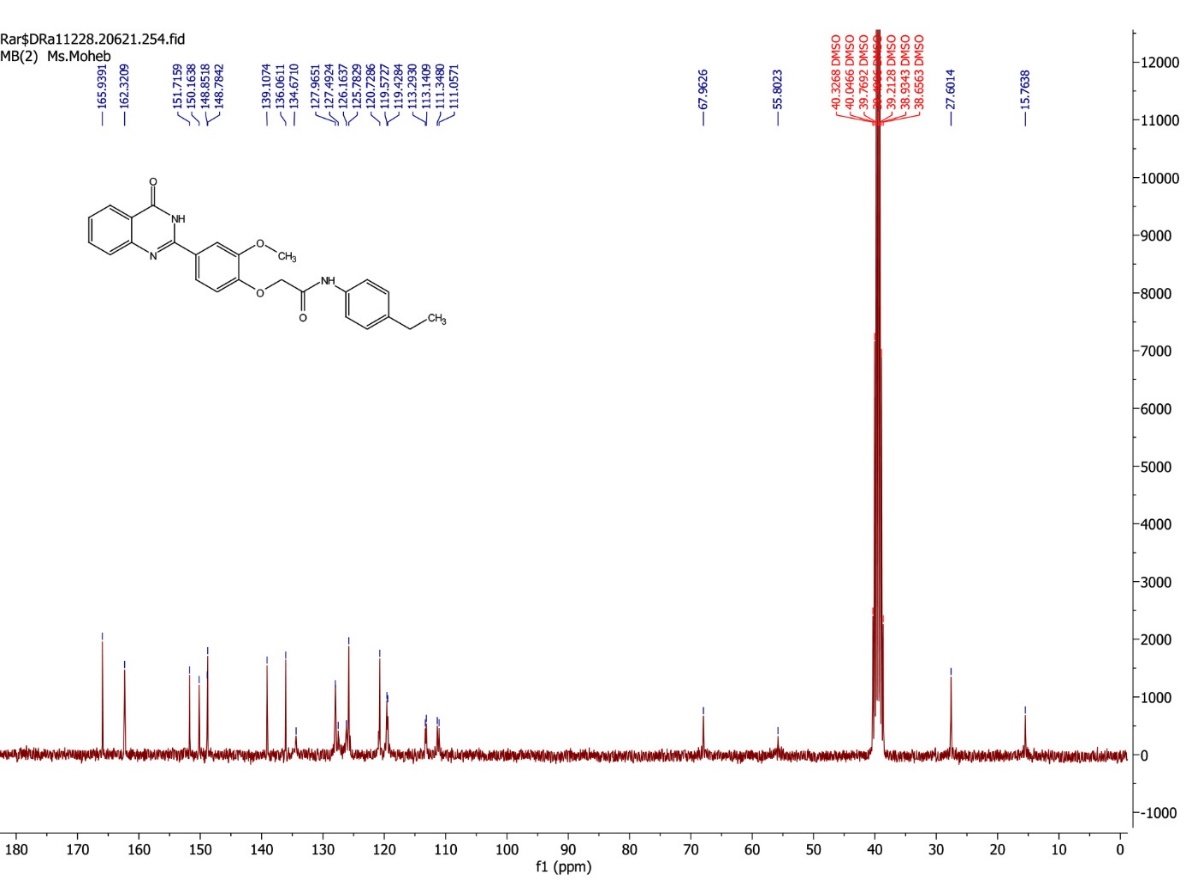


*Fig. S12 .2-(2-methoxy-4-(4-oxo-3,4-dihydroquinazolin-2-yl)phenoxy)-N-(4-methoxyphenyl)acetamide (****7l****)*


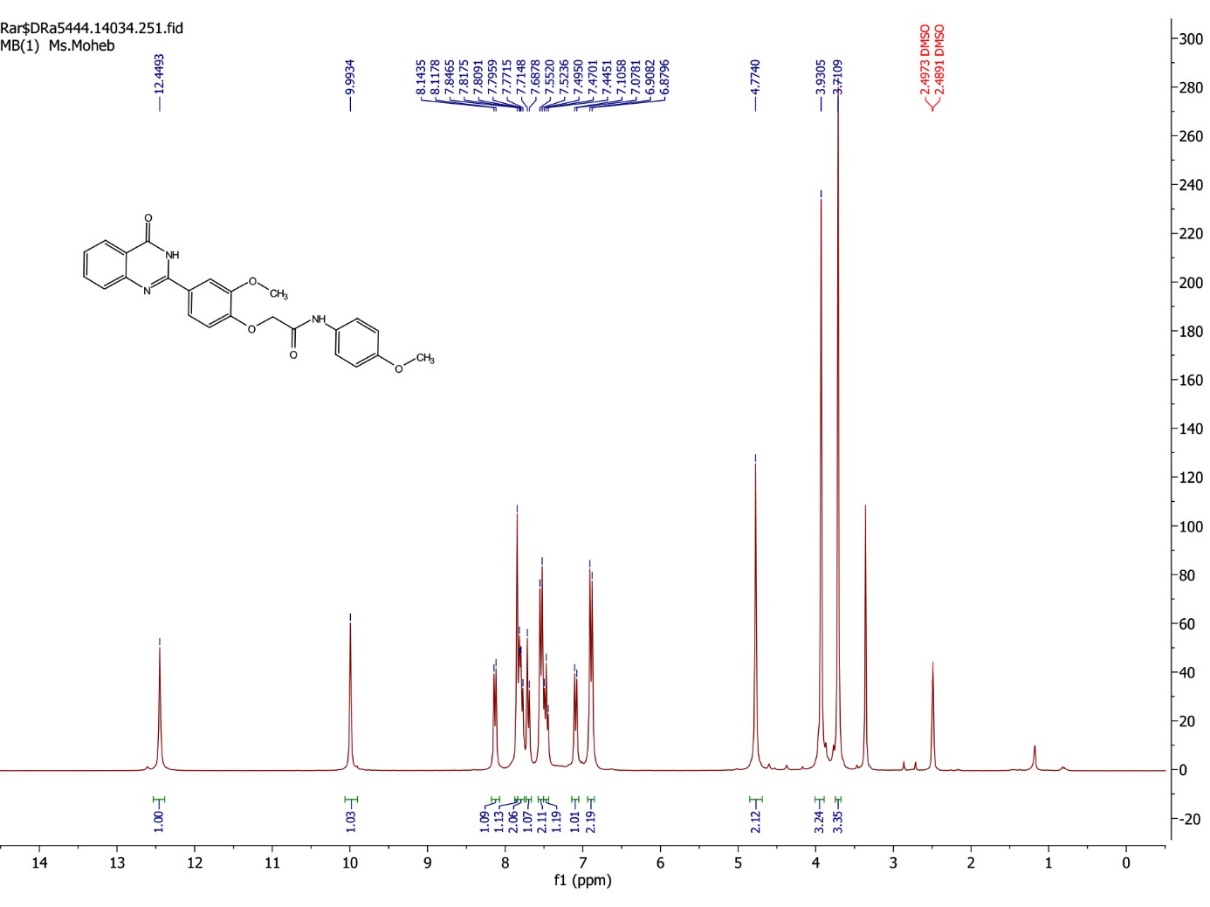


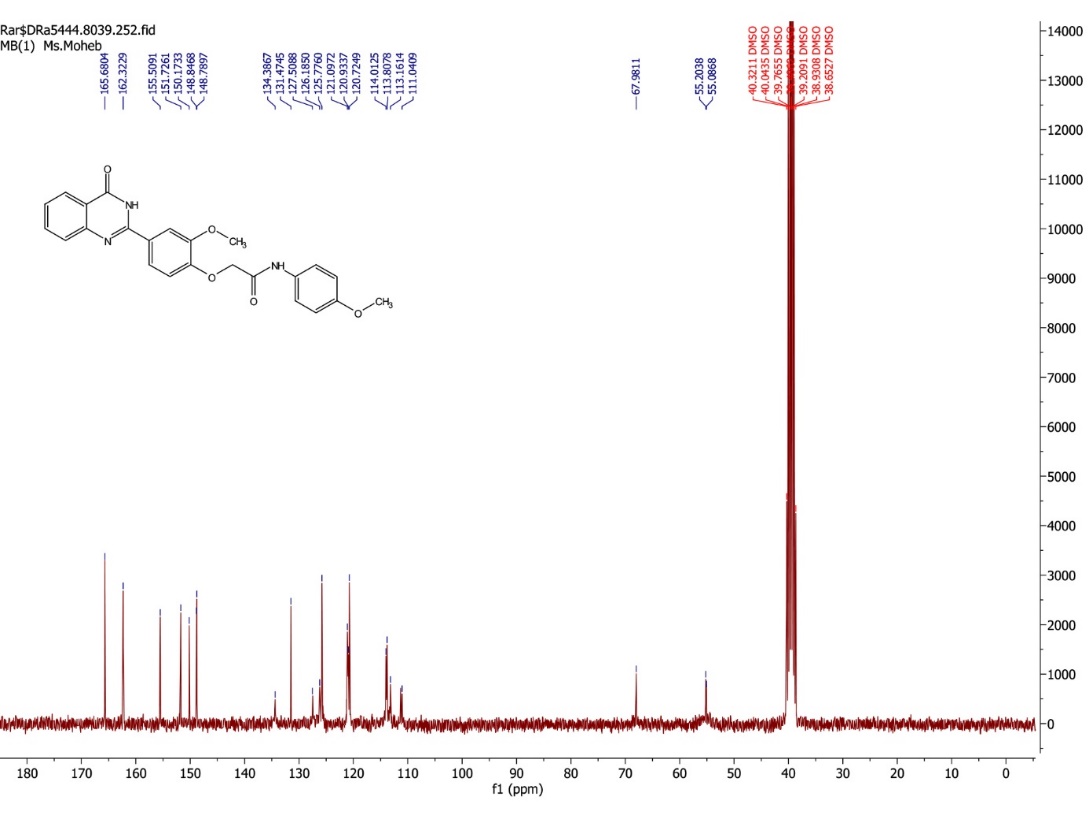


*Fig. S13.N-(4-hydroxyphenyl)-2-(2-methoxy-4-(4-oxo-3,4-dihydroquinazolin-2-yl)phenoxy)acetamide (****7m****)*


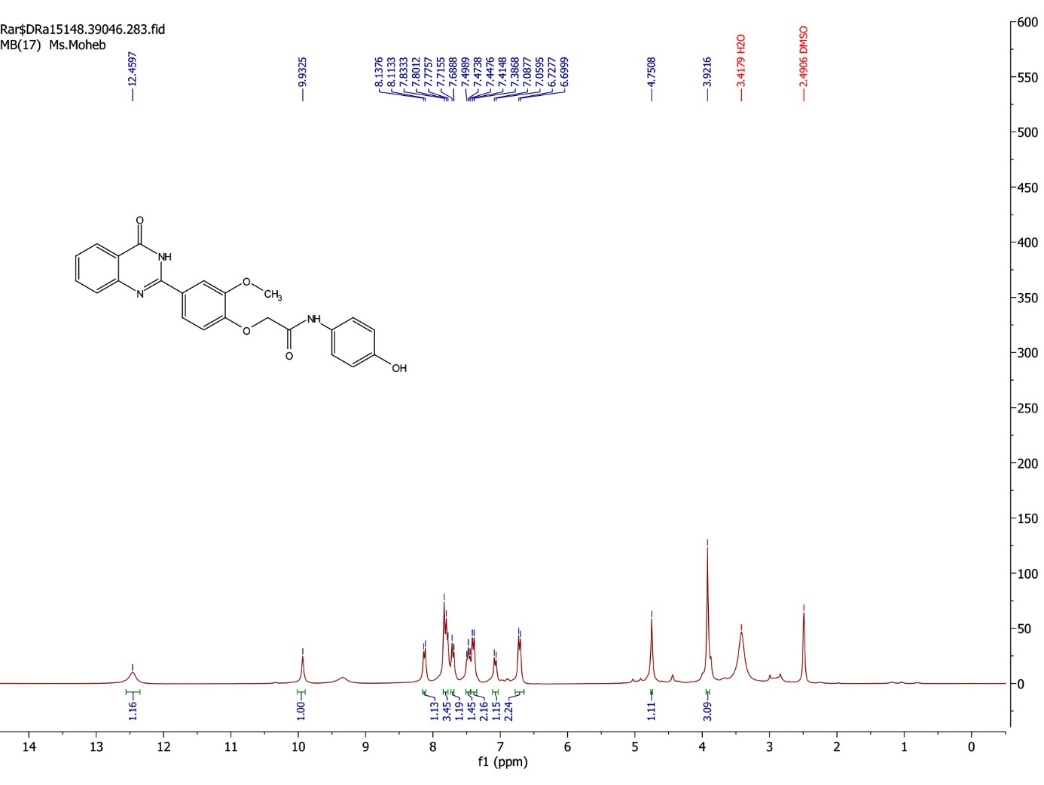


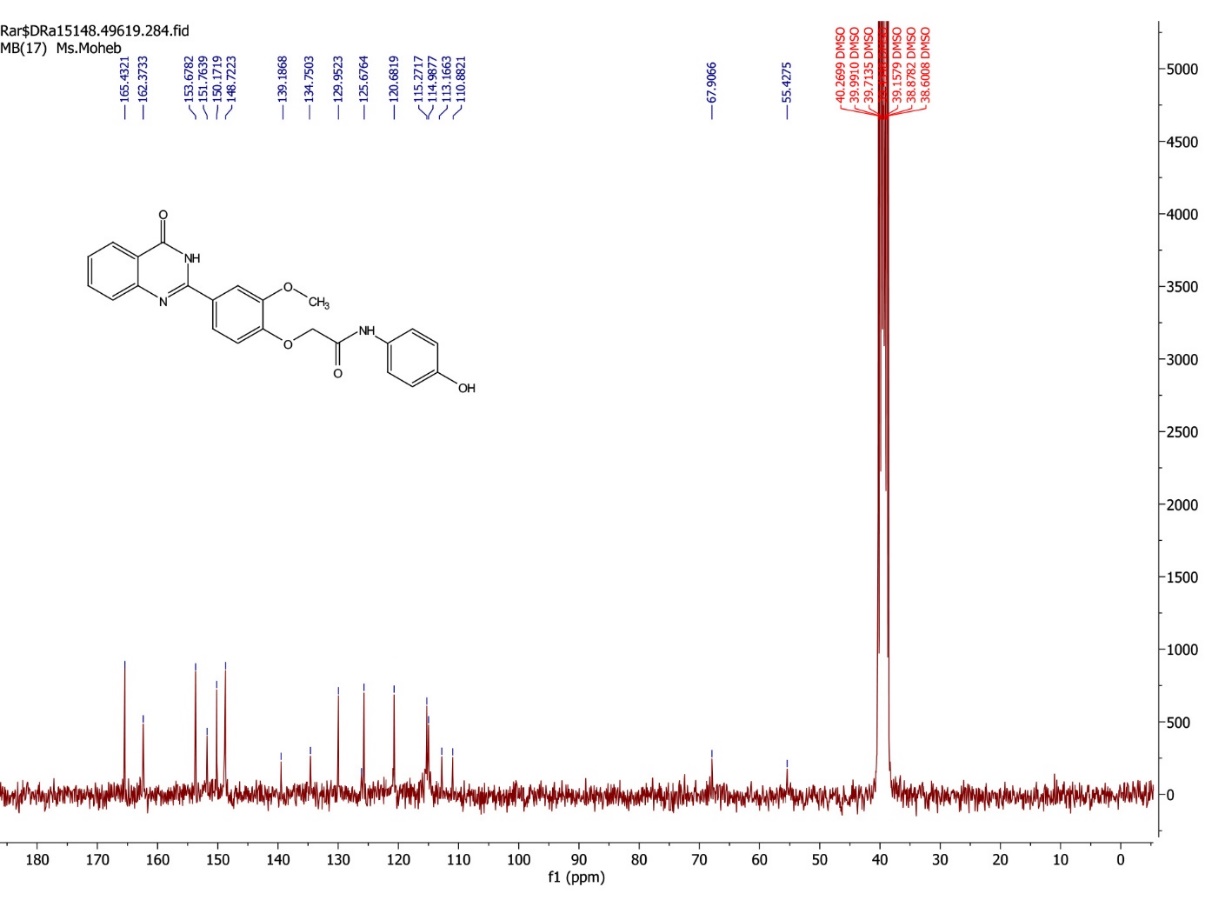


*Fig. S14.2-(2-methoxy-4-(4-oxo-3,4-dihydroquinazolin-2-yl)phenoxy)-N-(4-nitrophenyl)acetamide (****7n****)*


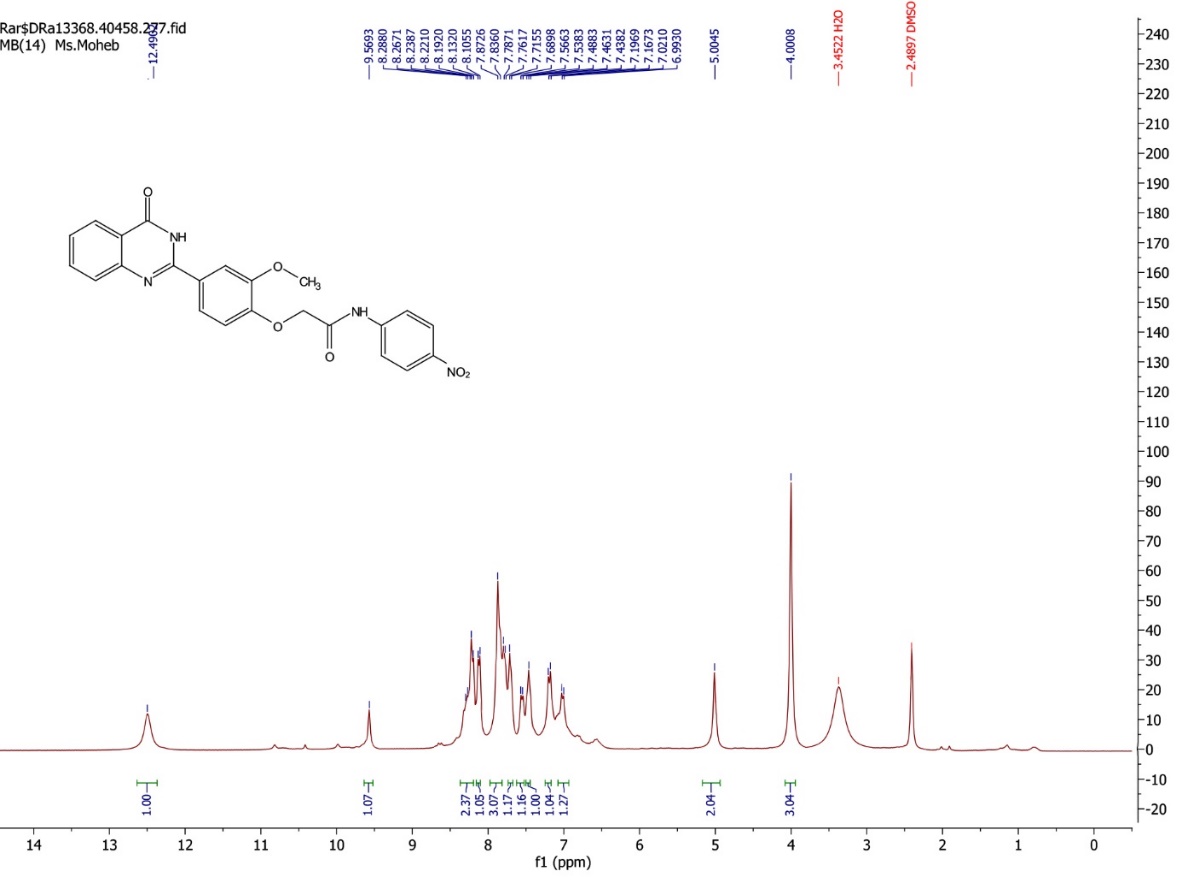


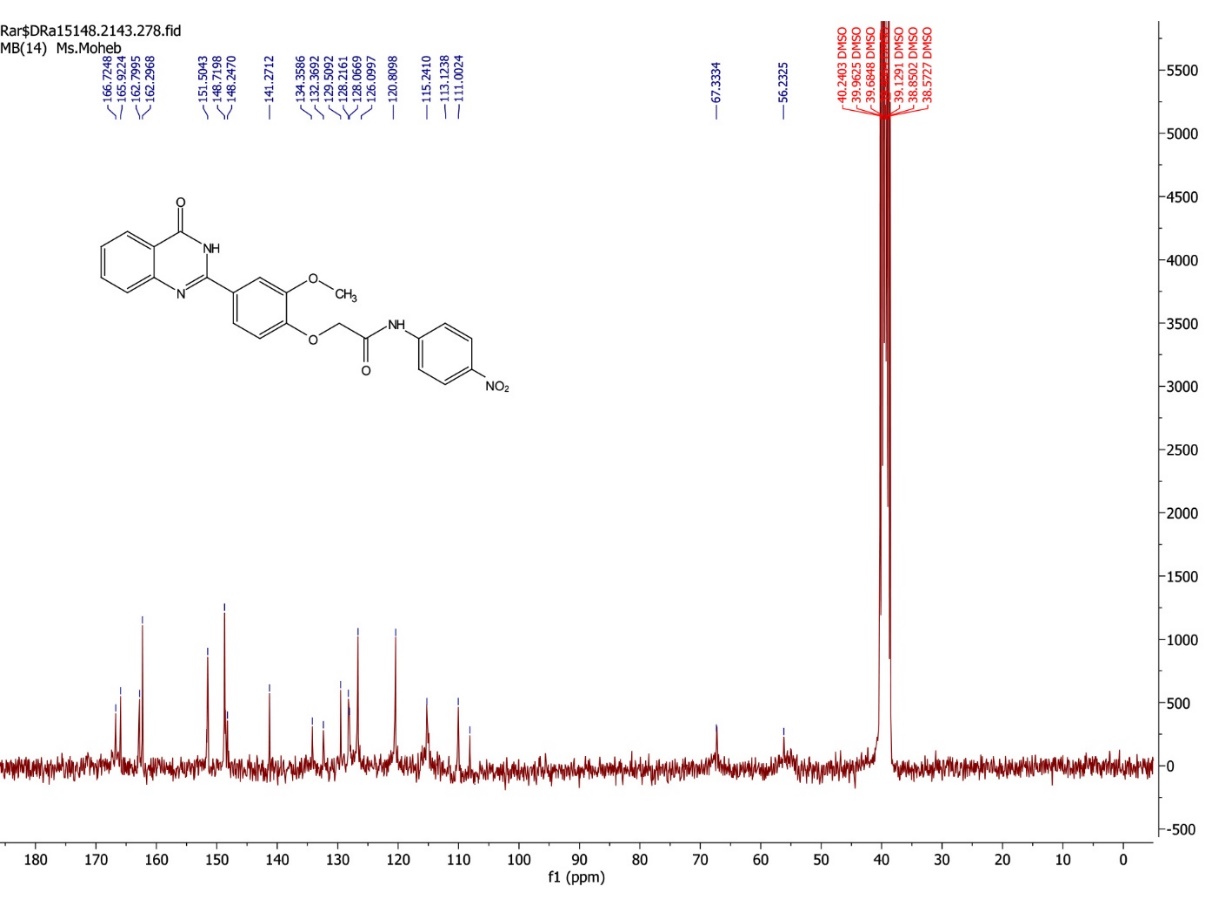


*Fig. S15.2-(2-methoxy-4-(4-oxo-3,4-dihydroquinazolin-2-yl)phenoxy)-N-(naphthalen-2-yl)acetamide (****7o****)*


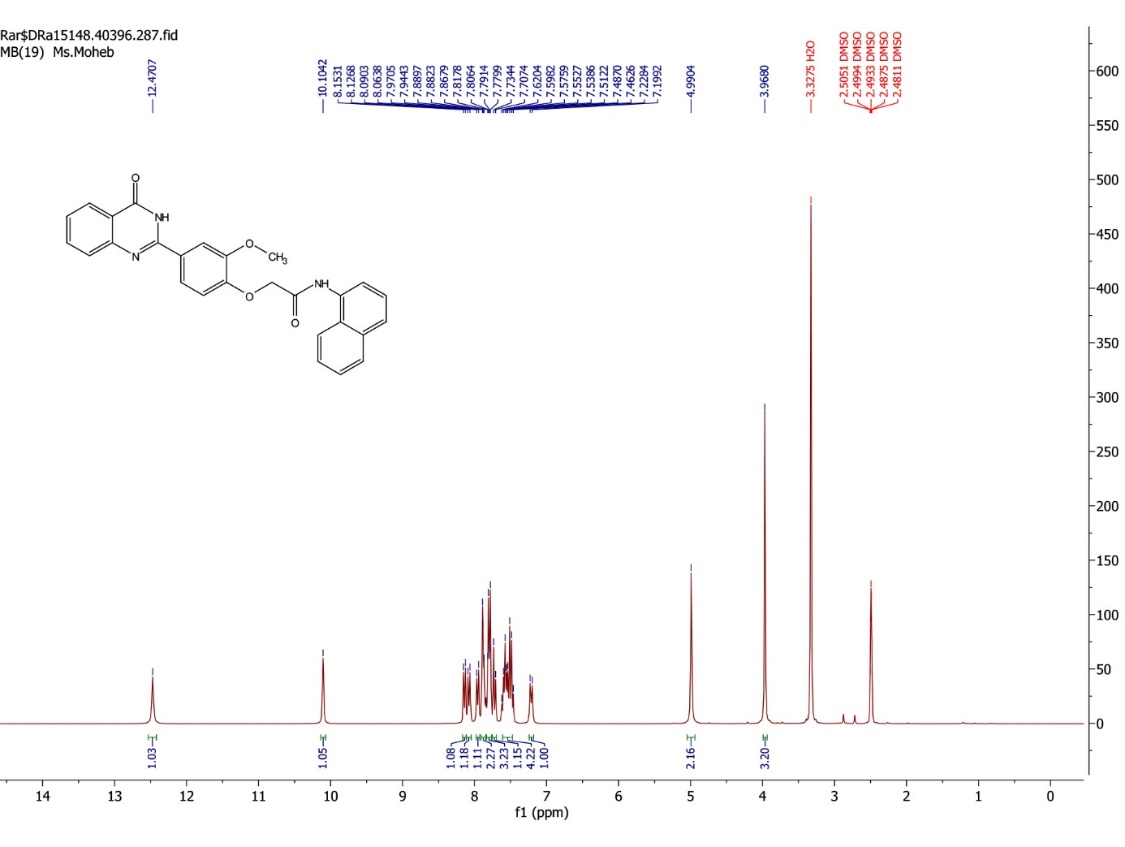


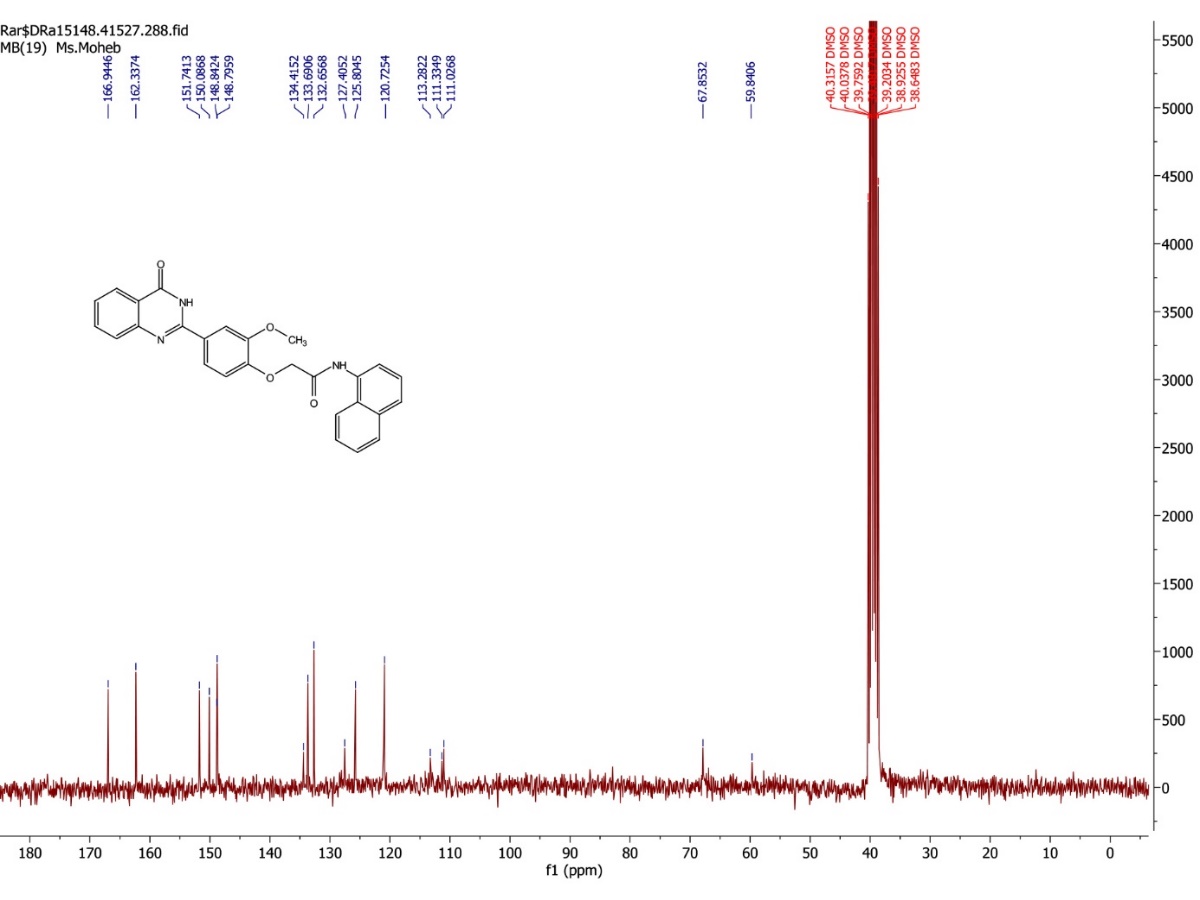


*Fig. S16.2-(2-methoxy-4-(4-oxo-3,4-dihydroquinazolin-2-yl)phenoxy)-N-(4-methylbenzyl)acetamide (****7p****)*


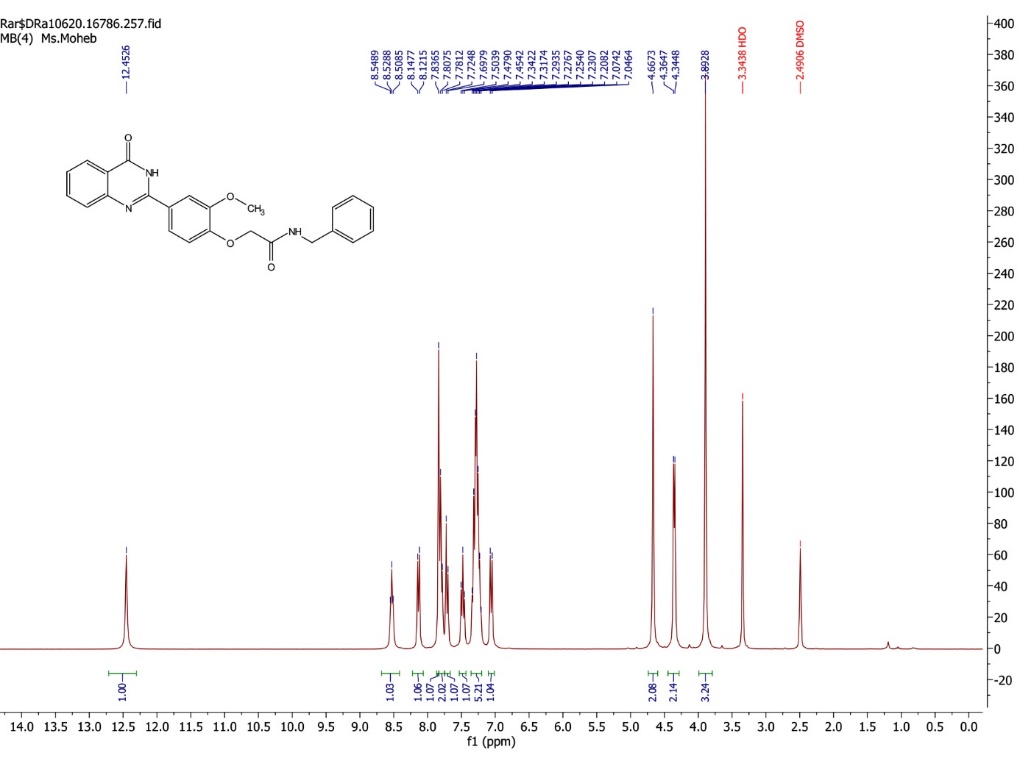


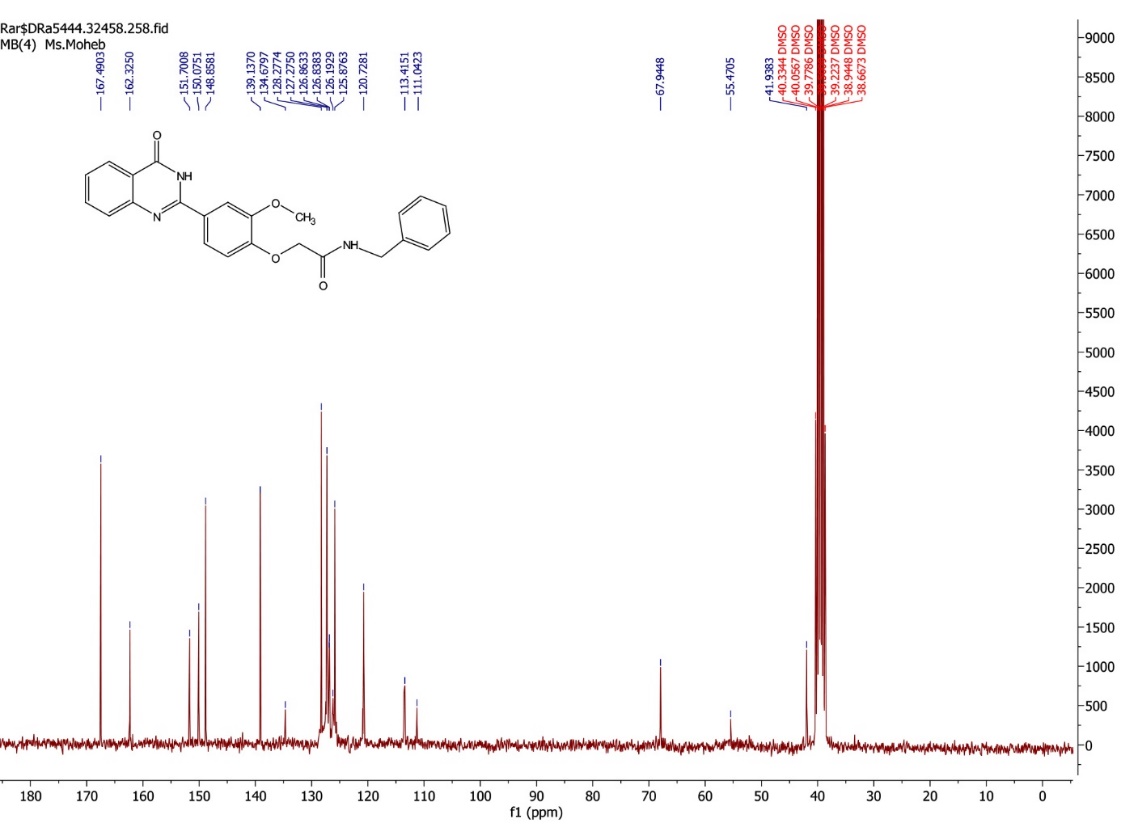


*Fig. S17. .N-(4-fluorobenzyl)-2-(2-methoxy-4-(4-oxo-3,4-dihydroquinazolin-2-yl)phenoxy)acetamide (****7q****)*


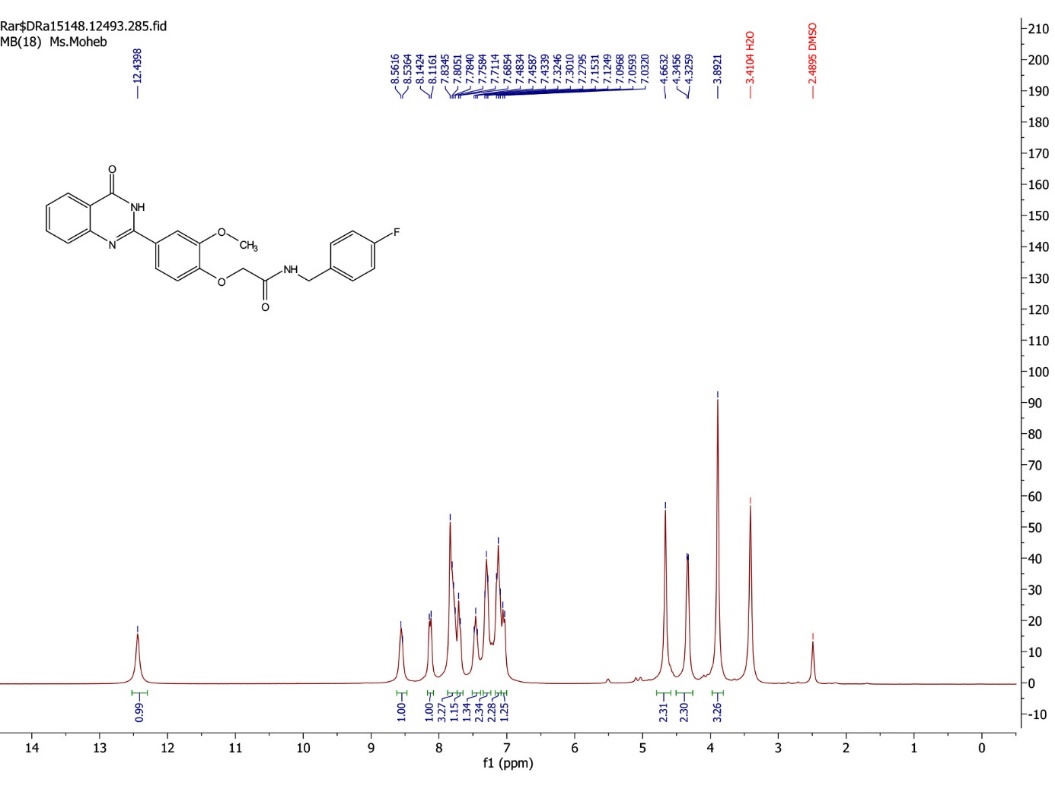


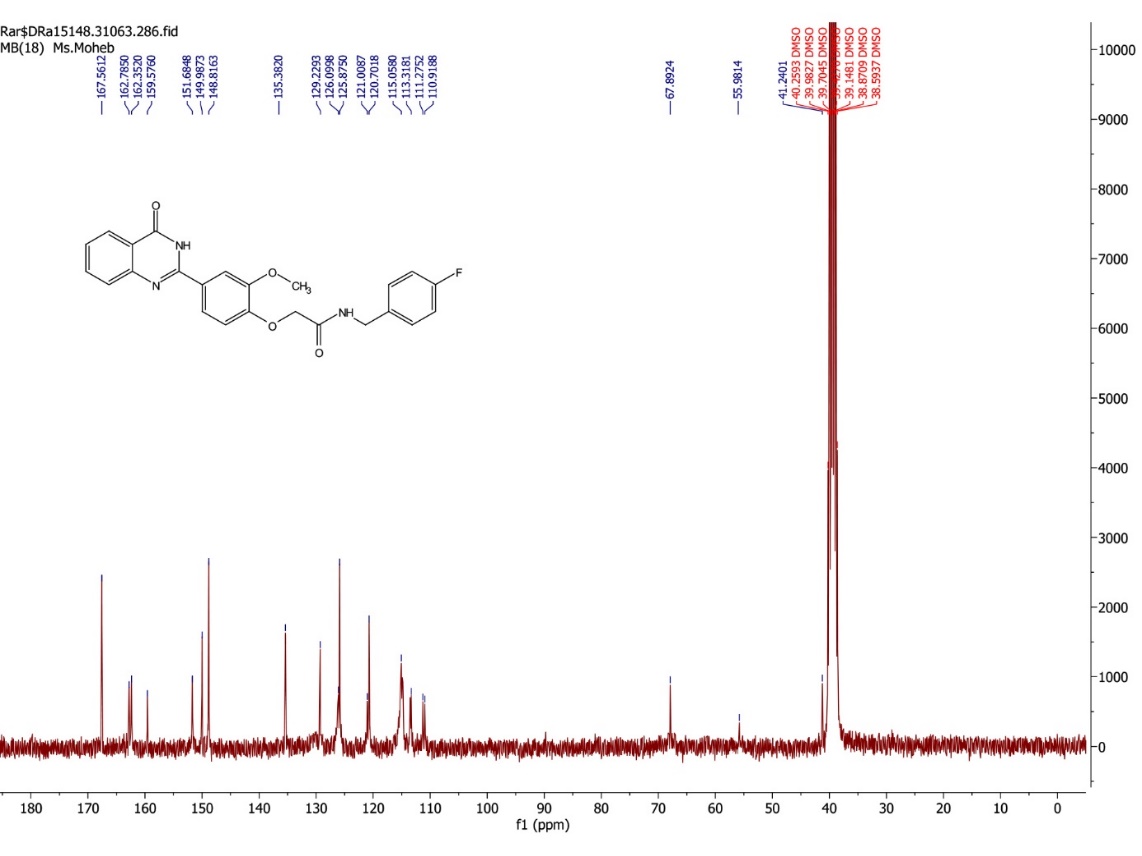


*Fig. S18. N-benzyl-2-(2-methoxy-4-(4-oxo-3,4-dihydroquinazolin-2-yl)phenoxy)acetamide (****7r****)*


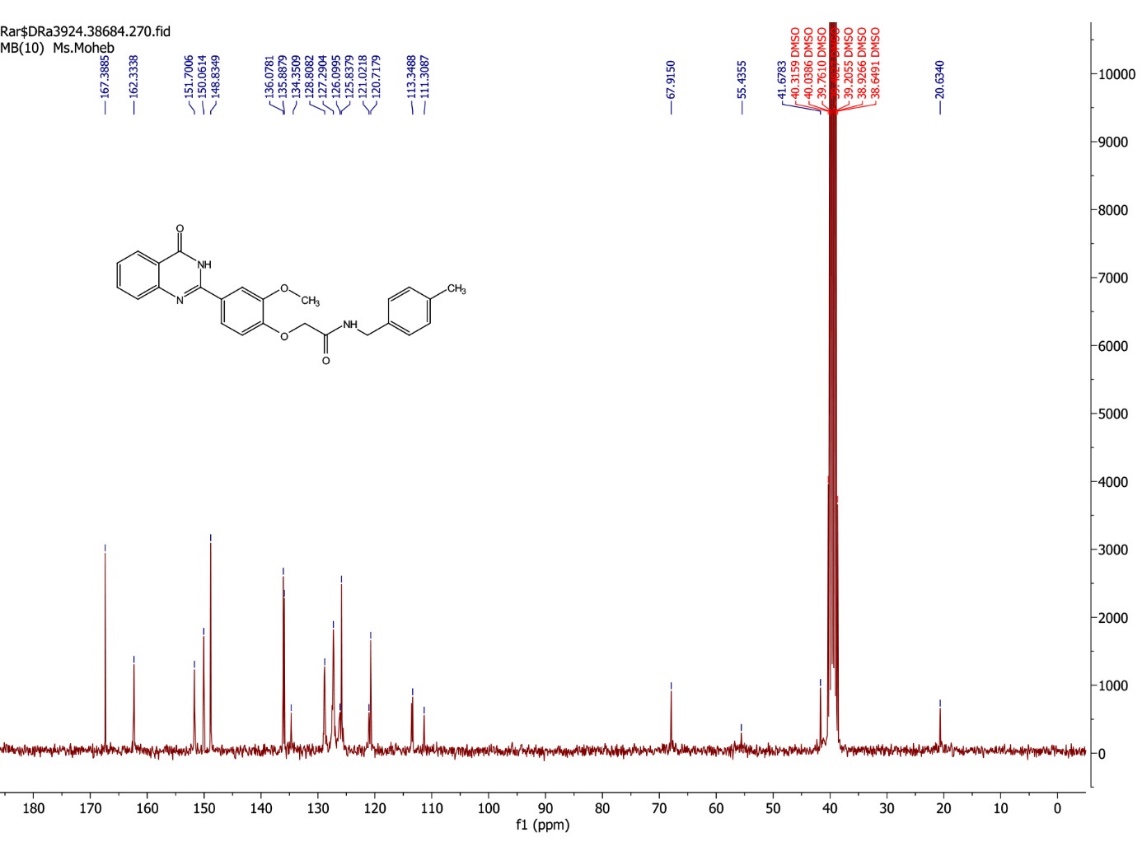


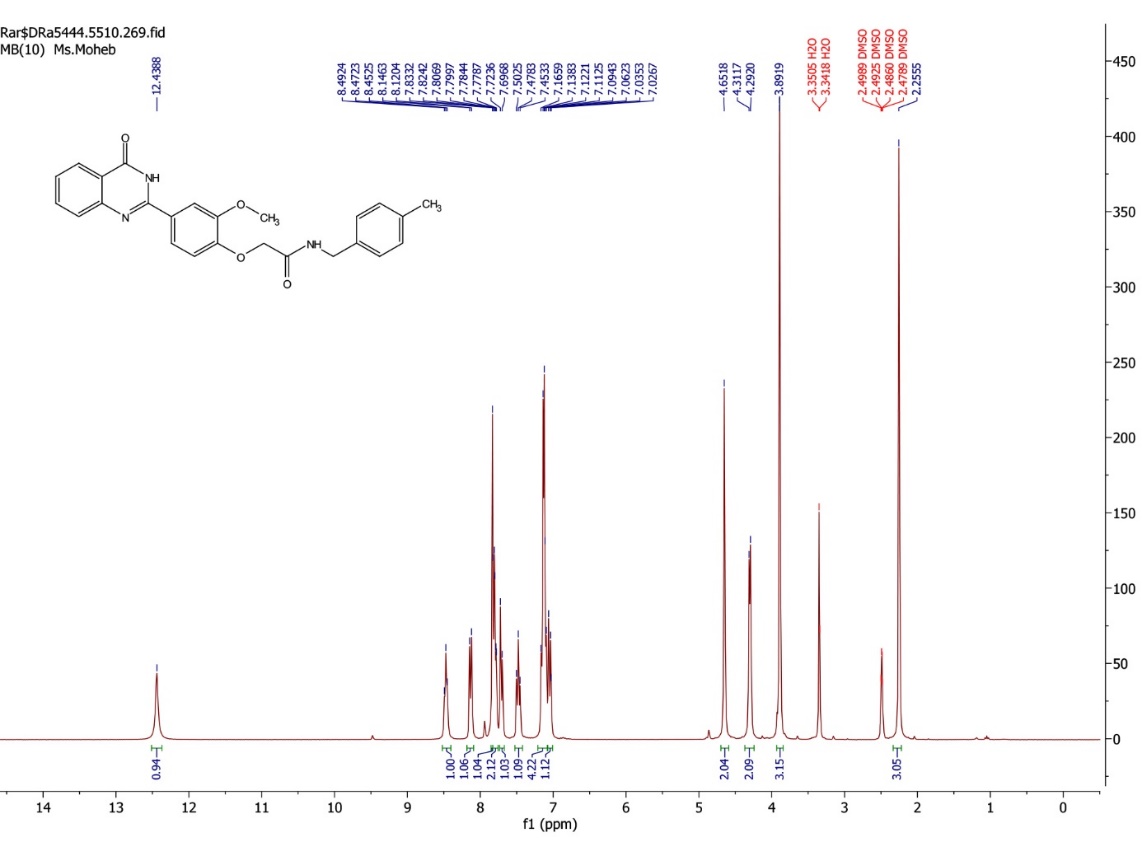

Supplement: Supplementary file 1 — Additional file 1: Figure S1. 2-(2-methoxy-4-(4-oxo-3,4-dihydroquinazolin-2-yl)phenoxy)-N-phenylacetamide (7a). Figure S2. N-(2-fluorophenyl)-2-(2-methoxy-4-(4-oxo-3,4-dihydroquinazolin-2-yl)phenoxy)acetamide (7b). Figure S3. N-(4-fluorophenyl)-2-(2-methoxy-4-(4-oxo-3,4-dihydroquinazolin-2-yl)phenoxy)acetamide (7c). Figure S4. N-(2-chlorophenyl)-2-(2-methoxy-4-(4-oxo-3,4-dihydroquinazolin-2-yl)phenoxy)acetamide (7d). Figure S5. N-(3-chlorophenyl)-2-(2-methoxy-4-(4-oxo-3,4-dihydroquinazolin-2-yl)phenoxy)acetamide (7e). Figure S6. N-(4-chlorophenyl)-2-(2-methoxy-4-(4-oxo-3,4-dihydroquinazolin-2-yl)phenoxy)acetamide (7f). Figure S7. N-(4-bromophenyl)-2-(2-methoxy-4-(4-oxo-3,4-dihydroquinazolin-2-yl)phenoxy)acetamide (7g). Figure S8. 2-(2-methoxy-4-(4-oxo-3,4-dihydroquinazolin-2-yl)phenoxy)-N-(o-tolyl)acetamide (7 h). Figure S9. 2-(2-methoxy-4-(4-oxo-3,4-dihydroquinazolin-2-yl)phenoxy)-N-(p-tolyl)acetamide (7i). Figure S10. N-(2,6-dimethylphenyl)-2-(2-methoxy-4-(4-oxo-3,4-dihydroquinazolin-2-yl)phenoxy)acetamide (7j). Figure S11. N-(4-ethylphenyl)-2-(2-methoxy-4-(4-oxo-3,4-dihydroquinazolin-2-yl)phenoxy)acetamide (7k). Figure S12. 2-(2-methoxy-4-(4-oxo-3,4-dihydroquinazolin-2-yl)phenoxy)-N-(4-methoxyphenyl)acetamide (7 l). Figure S13. N-(4-hydroxyphenyl)-2-(2-methoxy-4-(4-oxo-3,4-dihydroquinazolin-2-yl)phenoxy)acetamide (7m). Figure S14. 2-(2-methoxy-4-(4-oxo-3,4-dihydroquinazolin-2-yl)phenoxy)-N-(4-nitrophenyl)acetamide (7n). Figure S15. 2-(2-methoxy-4-(4-oxo-3,4-dihydroquinazolin-2-yl)phenoxy)-N-(naphthalen-2-yl)acetamide (7o). Figure S16. 2-(2-methoxy-4-(4-oxo-3,4-dihydroquinazolin-2-yl)phenoxy)-N-(4-methylbenzyl)acetamide (7p). Figure S17. N-(4-fluorobenzyl)-2-(2-methoxy-4-(4-oxo-3,4-dihydroquinazolin-2-yl)phenoxy)acetamide (7q). Figure S18. N-benzyl-2-(2-methoxy-4-(4-oxo-3,4-dihydroquinazolin-2-yl)phenoxy)acetamide (7r). [file 13065_2022_885_MOESM1_ESM.docx]
